# Zinc transport *via* ZNT5-6 and ZNT7 is critical for cell surface glycosylphosphatidylinositol-anchored protein expression

**DOI:** 10.1016/j.jbc.2022.102011

**Published:** 2022-05-04

**Authors:** Takumi Wagatsuma, Keiko Shimotsuma, Akiko Sogo, Risa Sato, Naoya Kubo, Sachiko Ueda, Yasuo Uchida, Masato Kinoshita, Taiho Kambe

**Affiliations:** 1 Division of Integrated Life Science, Graduate School of Biostudies, Kyoto University, Kyoto, Japan; 2 Division of Applied Biosciences, Graduate School of Agriculture, Kyoto University, Kyoto, Japan; 3 Division of Membrane Transport and Drug Targeting, Graduate School of Pharmaceutical Sciences, Tohoku University, Sendai, Japan

**Keywords:** zinc, glycosylphosphatidylinositol (GPI anchor), transporter, cell surface, ER quality control, ZNT, early secretory pathway, ectoenzyme, phosphatidylinositol glycan anchor biosynthesis (PIG), ACE, angiotensin-converting enzyme, ALP, alkaline phosphatase, cDNA, complementary DNA, CP, ceruloplasmin, DKO, double KO, ER, endoplasmic reticulum, ERAD, ER-associated protein degradation, gACE, germline specific ACE, GPI, glycosylphosphatidylinositol, HA, hemagglutinin, IgG, immunoglobulin G, IRES, internal ribosome entry site, LHA, left homology arm, PIG, phosphatidylinositol glycan anchor biosynthesis, PLAP, placental ALP, RHA, right homology arm, sACE, somatic ACE, SWATH-MS, sequential window acquisition of all theoretical fragment ion spectra mass spectrometry, TKO, triple KO, TNAP, tissue nonspecific ALP, ZNT, Zn transporter

## Abstract

Glycosylphosphatidylinositol (GPI)-anchored proteins play crucial roles in various enzyme activities, cell signaling and adhesion, and immune responses. While the molecular mechanism underlying GPI-anchored protein biosynthesis has been well studied, the role of zinc transport in this process has not yet been elucidated. Zn transporter (ZNT) proteins mobilize cytosolic zinc to the extracellular space and to intracellular compartments. Here, we report that the early secretory pathway ZNTs (ZNT5–ZNT6 heterodimers [ZNT5-6] and ZNT7–ZNT7 homodimers [ZNT7]), which supply zinc to the lumen of the early secretory pathway compartments are essential for GPI-anchored protein expression on the cell surface. We show, using overexpression and gene disruption/re-expression strategies in cultured human cells, that loss of ZNT5-6 and ZNT7 zinc transport functions results in significant reduction in GPI-anchored protein levels similar to that in mutant cells lacking phosphatidylinositol glycan anchor biosynthesis (*PIG*) genes. Furthermore, medaka fish with disrupted *Znt5* and *Znt7* genes show touch-insensitive phenotypes similar to zebrafish *Pig* mutants. These findings provide a previously unappreciated insight into the regulation of GPI-anchored protein expression and protein quality control in the early secretory pathway.

Approximately one-third of all cellular proteins encounter the endoplasmic reticulum (ER) secretory pathway (1), resulting in posttranslational modifications such as glycosylation, disulfidation, and glycosylphosphatidylinositol (GPI) modification at the C terminus producing GPI-anchored protein. There are more than 150 characterized GPI-anchored proteins in humans (2, 3, 4), which function as enzymes, receptors, adhesion proteins, and complement regulatory proteins on the cell surface (5). The biosynthesis of GPI-anchored proteins requires more than 20 phosphatidylinositol glycan anchor biosynthesis (PIG) proteins involving sequential additions of sugar and other components to phosphatidylinositol in multiple reaction steps. Thus, mutations in multiple *PIG* genes are associated with defects in GPI-anchored proteins (inherited GPI deficiency) resulting in prominent human diseases including intellectual disability, hypotonia, facial dysmorphism, seizures, and dystonia (6). The effect of reduced GPI-anchored protein expression requires further investigation.

A considerable proportion of the secretome requires zinc before reaching their final destinations (7, 8, 9). Zn transporter 5 (ZNT5)–ZNT6 (encoded by *SLC30A5* and *SLC30A6*) heterodimers and ZNT7–ZNT7 (encoded by *SLC30A7*) homodimers (hereafter, ZNT5-6 and ZNT7) are zinc entry routes into the compartments of the early secretory pathway and play pivotal roles in homeostatic maintenance of secretory pathway function (10, 11, 12). They activate zinc ectoenzymes (secretory and extracellularly localized zinc-requiring enzymes) through zinc metalation at their active sites (10, 13, 14, 15, 16). Some zinc ectoenzymes are degraded in cells lacking ZNT5-6 and ZNT7, while others are activated in the same cells without degradation, therefore independent of ZNT5-6 and ZNT7 (15, 17). However, the causes of these differences are unknown (18). Recently, we found that the secretory form of placental alkaline phosphatase (secPLAP, also known as secALPP) zinc ectoenzyme is stably expressed in chicken DT40 cells lacking ZNT5-6 and ZNT7, whereas PLAP is not, although both are inactive (16). This indicates that protein stability differences in zinc ectoenzyme in cells lacking ZNT5-6 and ZNT7 are independent of zinc metalation.

In this study, the ZNT5-6 and ZNT7 function was analyzed *via* mutational manipulation studies. The GPI-anchored protein expression was impaired in mutant *Z5Z7*–double KO (DKO) cells where ZNT5-6 and ZNT7 are nonfunctional, as confirmed *via* sequential window acquisition of all theoretical fragment ion spectra mass spectrometry (SWATH-MS) analysis (19) compared with the WT. Moreover, disruption of both *ZNT5* and *ZNT7* led to similar phenotypes to those caused by *PIG* gene disruptions in the cells and in medaka fish (*Oryzias latipes*). The mutant medaka exhibited touch-insensitive phenotypes, which are similar to those of zebrafish *Pigk* and *Pigu* mutants (20, 21). Our results provide insights into the biological functions of zinc mediated by ZNT5-6 and ZNT7 within the compartments of the early secretory pathway and improve our understanding of protein quality control in the ER and the secretory pathway *via* GPI-anchored protein synthesis.

## Results

### ZNT5-6 and ZNT7 are indispensable for zinc ectoenzyme expression modified by the GPI-anchor

**Figure 1:**
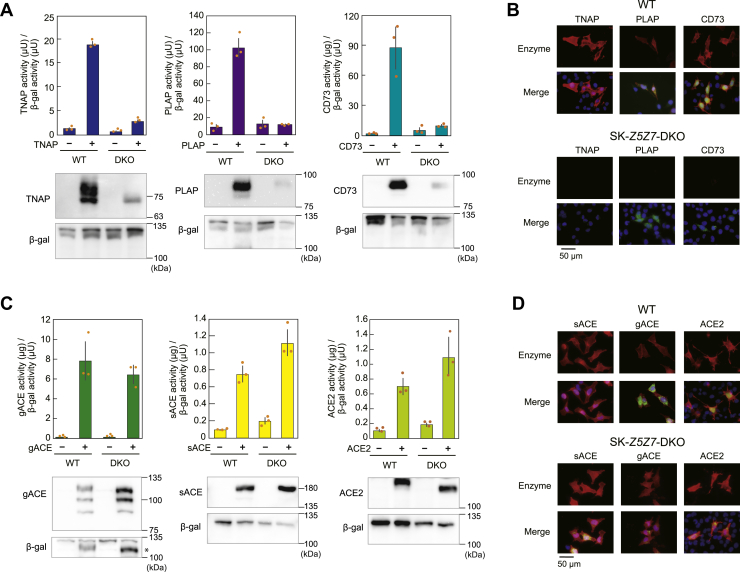
**Properties of transiently expressed GPI-anchored and single membrane–spanning polypeptide-anchored zinc ectoenzymes in *Z5Z7*-DKO cells.***A*, the activity and protein expression of GPI-anchored zinc ectoenzymes (TNAP, PLAP, and CD73). *B*, immunofluorescence staining of TNAP, PLAP, and CD73, detected in red fluorescence. *C*, the activity and protein expression of single membrane–spanning polypeptide-anchored zinc ectoenzymes (sACE, gACE, and ACE2). gACE, detected in the *lower left panel* for the blotting of β-gal, is indicated with an *asterisk* (∗). *D*, immunofluorescence staining of sACE, gACE, and ACE2. In (*A*) and (*C*), expression plasmids were transiently transfected in WT A549 cells and A549-*Z5Z7*-DKO cells. β-galactosidase (β-gal) was used as the internal control. All activities are expressed as the mean ± SD of triplicate experiments (*upper graphs*). Ten micrograms of total cellular lysate prepared from transiently transfected cells was subjected to immunoblot analysis (*lower panels*). In (*B*) and (*D*), WT SK-MEL-2 and SK-*Z5Z7*-DKO cells were transiently transfected with expression plasmid harboring each cDNA in IRES-GFP plasmid, and transfected cells were discriminated by GFP fluorescence. Merged images with GFP and DAPI are shown. Each experiment was performed at least three times and representative results from independent experiments are shown. ACE, angiotensin-converting enzyme; cDNA, complementary DNA; DAPI, 4,6-diamino-2-phenylindole; DKO, double KO; gACE, germline specific ACE; GPI, glycosylphosphatidylinositol; IRES, internal ribosome entry site; PLAP, placental ALP; sACE, somatic ACE; TNAP, tissue nonspecific ALP.

Human A549 cells and SK-MEL-2 cells deficient in both *ZNT5* and *ZNT7* (A549-*Z5Z7*-DKO and SK-*Z5Z7*-DKO) were generated for this work (Table 1). Transient transfection of plasmids expressing GPI-anchored zinc ectoenzymes (tissue nonspecific alkaline phosphatase [ALP] [TNAP, also known as ALPL]), PLAP, and CD73 (also known as NT5E) in WT A549 cells and A549-*Z5Z7*-DKO cells showed substantially decreased activity and protein expression in A549-*Z5Z7*-DKO cells compared with those in WT A549 cells (Fig. 1*A*). Immunofluorescence staining failed to detect their cell surface expression in SK-*Z5Z7*-DKO cells, while GFP using internal ribosome entry site (IRES)–GFP plasmid showed coexpression (Fig. 1*B*). Meanwhile, the activity and cell surface expression of transiently expressed C-terminal single membrane–spanning polypeptide-anchored zinc ectoenzymes, somatic angiotensin-converting enzyme (sACE), germline-specific half-size splicing variant of sACE (gACE) (22, 23), and ACE2 was comparable between WT A549 cells and A549-*Z5Z7*-DKO cells (Fig. 1, *C* and *D*). These results suggest that the GPI-anchor is a critical determinant of specific zinc ectoenzyme expression.

**Table 1:** Mutations introduced in *ZNT5*, *ZNT7*, and *PIG* genes

Cells	Target region	Mutation
A549-*Z5Z7*-DKO	Exon 11 in *ZNT5*, exon 2 in *ZNT7*	1-bp deletion in both alleles in *ZNT5*, 7-bp or 9-bp deletion in *ZNT7*
SK-*Z5Z7*-DKO	Exon 11 in *ZNT5*, exon 2 in *ZNT7*	13-bp or 18-bp deletion in *ZNT5*, 1-bp or 7-bp insertion in *ZNT7*
HAP-*PIGN*-KO	Exon 4 in *PIGN*	8-bp deletion
HAP-*PIGO*-KO	Exon 4 in *PIGO*	1-bp deletion
HAP-*PIGG*-KO	Exon 2 in *PIGG*	283-bp insertion
HAP-*PIGNPIGOPIGG*-TKO	Exon 4 in *PIGN*, Exon 4 in *PIGO*, exon 2 in *PIGG*	8-bp deletion in *PIGN*, 1-bp insertion in *PIGO*, 33-bp deletion in *PIGG*
HAP-*PIGA*-KO	Exon 3 in *PIGA*	1-bp deletion
HAP-*PIGT*-KO	Exon 2 in *PIGT*	1-bp insertion
HAP-*Z5Z7PIGG*-TKO	Exon 11 in *ZNT5*, exon 2 in *ZNT7*, exon 2 in *PIGG*	2-bp deletion in *ZNT5*, 28-bp deletion in *ZNT7*, 21-bp deletion in *PIGG*
HAP-*Z5Z7PIGN*-TKO	Exon 11 in *ZNT5*, exon 2 in *ZNT7*, exon 4 in *PIGN*	2-bp deletion in *ZNT5*, 28-bp deletion in *ZNT7*, 1-bp deletion in *PIGN*
HAP-*Z5Z7PIGA*-TKO	Exon 11 in *ZNT5*, exon 2 in *ZNT7*, exon 3 in *PIGA*	3-bp deletion in *ZNT5*, 9-bp deletion in *ZNT7*, 1-bp deletion in *PIGA*
HAP-*Z5Z7PIGT*-TKO	Exon 11 in *ZNT5*, exon 2 in *ZNT7*, exon 2 in *PIGT*	2-bp deletion in *ZNT5*, 28-bp deletion in *ZNT7*, 1-bp insertion in *PIGT*

Information regarding HAP-*Z5Z7*-DKO is described in ref. (16).

**Figure 2:**
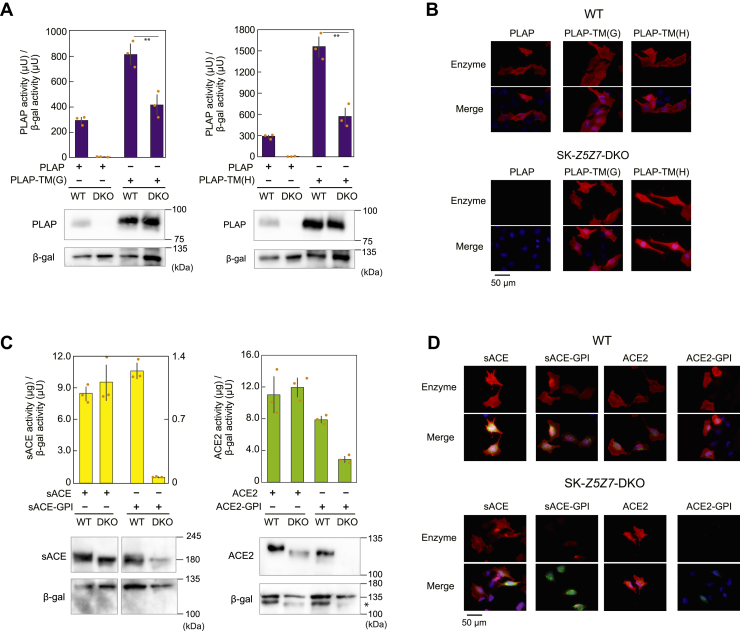
**Properties of chimeric zinc ectoenzyme expression in *Z5Z7*-DKO cells.***A*, activity and protein expression of chimeric PLAP mutants (PLAP-TM(H) or PLAP-TM(G)) in which the GPI anchor was substituted with a single membrane–spanning polypeptide anchor. *B*, immunofluorescence staining of transiently expressed PLAP-TM(H) or PLAP-TM(G). *C*, activity and protein expression of chimeric mutants of sACE and ACE2 (sACE-GPI or ACE2-GPI), in which the single membrane–spanning polypeptide-anchor was substituted with the GPI-anchor. In the *left graph*, sACE activity is shown on the *left-hand axis* of ordinate, while sACE-GPI activity is shown on the *right-hand axis*. ACE2, detected in the *lower right panel* for the blotting of β-gal, is indicated by an *asterisk* (∗). *D*, immunofluorescence staining of transiently expressed ACE-GPI or ACE2-GPI. In (*A*) and (*C*), expression plasmids were transiently transfected in WT A549 cells and A549-*Z5Z7*-DKO cells. In (*B*) and (*D*), WT SK-MEL-2 and SK-*Z5Z7*-DKO cells were transiently transfected with expression plasmid harboring each cDNA in IRES-GFP plasmid. Each experiment was performed at least three times, and representative results from independent experiments are shown. ACE, angiotensin-converting enzyme; cDNA, complementary DNA; DKO, double KO; gACE, germline specific ACE; GPI, glycosylphosphatidylinositol; IRES, internal ribosome entry site; PLAP, placental ALP; sACE, somatic ACE.

This was examined in more detail using chimeric mutants of PLAP (PLAP-TM(H) and PLAP-TM(G)) wherein the C-terminal GPI anchor attachment portion of PLAP was substituted with a single membrane–spanning polypeptide-anchored portion of the cytosolic portion of influenza hemagglutinin (HA) or vesicular stomatitis virus glycoprotein since both mutants were located on the cell surface (24). PLAP-TM(H) and PLAP-TM(G) expression in A549-*Z5Z7*-DKO cells was comparable with that in WT A549 cells, although their activity was significantly decreased (Fig. 2*A*). Both proteins were localized to the cell surface when expressed in SK-*Z5Z7*-DKO and WT SK-MEL-2 (Fig. 2*B*). For comparison, chimeric sACE and ACE2 mutants were constructed by substituting their membrane-spanning polypeptide portion with the GPI attachment of PLAP to form ACE-GPI and ACE2-GPI, respectively. The activity and protein expression of both ACE-GPI and ACE2-GPI was substantially decreased in A549-*Z5Z7*-DKO cells compared with those in WT A549 cells (Fig. 2*C*). ACE-GPI and ACE2-GPI failed to localize to the cell surface when expressed in SK-*Z5Z7*-DKO cells (Fig. 2*D*). These results indicate that the GPI anchor is involved in the impairment of the activation and protein expression of GPI-anchored zinc ectoenzymes and that ZNT5-6 and ZNT7 play pivotal roles during this process.

The secretory forms of PLAP (secPLAP-HA) and ACE (secACE-HA) were constructed using an HA-tag in place of the C-terminal portion containing the GPI anchor attachment site and C-terminal single membrane–spanning polypeptide anchor, respectively. Both zinc ectoenzymes were detected in the spent medium of transfected WT A549 cells and A549-*Z5Z7*-DKO cells. However, secPLAP activity substantially decreased, while secACE activity was partially activated in A549-*Z5Z7*-DKO cells, compared with that in WT A549 cells (Fig. S1, *A* and *B*). These results indicate that the GPI anchor is responsible for the impairment of GPI-anchored zinc ectoenzyme expression in *Z5Z7*-DKO cells.

### GPI-anchored protein expression decreased in *Z5Z7*-DKO cells

**Figure 3:**
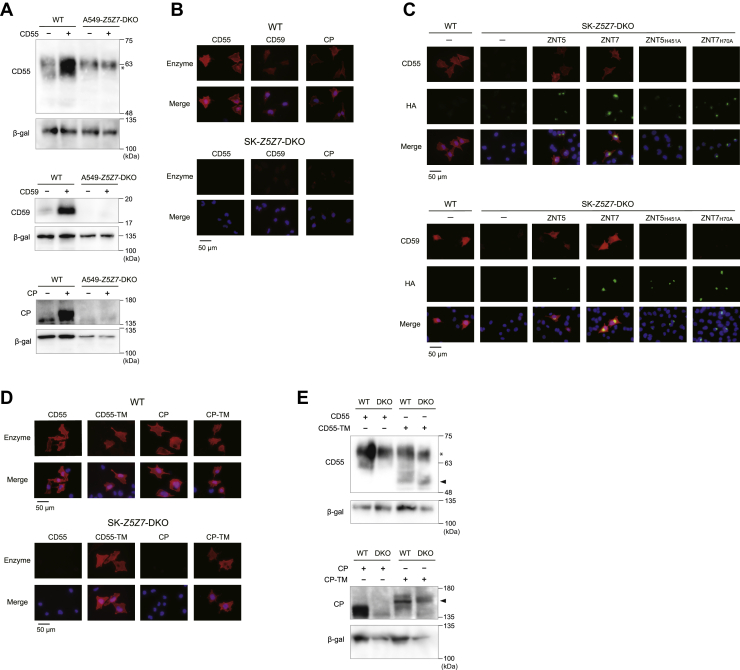
**Properties of GPI-anchored proteins transiently expressed in *Z5Z7*-DKO cells.***A*, protein expression of CD55, CD59, and CP in A549-*Z5Z7*-DKO cells and WT A549 cells. *B*, immunofluorescence staining of CD55, CD59, and CP in SK-*Z5Z7*-DKO cells and WT SK-MEL-2 cells. *C*, restoration of CD55 and CD59 expression on the cell surface in SK-*Z5Z7*-DKO cells by simultaneous expression of HA-ZNT5 or HA-ZNT7 but not by zinc transport–incompetent HA-ZNT5_H451A_ or HA-ZNT7_H70A_. Cells transfected with zinc transport incompetent ZNT5 or ZNT7 expression plasmids were cultured in the presence of 75 μM ZnSO_4_. *D* and *E*, expression of chimeric CD55 and CP proteins in which the GPI anchor was substituted with a single membrane–spanning polypeptide anchor was examined using immunofluorescence staining (*D*) and immunoblotting (*E*). Immunofluorescence staining and immunoblotting were performed in the same method as described in Figure 1. In (*E*), *arrowheads* indicate the position of CD-55-TM or CP-TM. Nonreducing SDS-PAGE was performed to detect CD55 in (*A*) and (*E*). A nonspecific band is indicated by an *asterisk* (∗). Each experiment was performed at least three times and representative results from independent experiments are shown. CP, ceruloplasmin; DKO, double KO; GPI, glycosylphosphatidylinositol; HA, hemagglutinin.

We then examined whether the GPI anchor is responsible for the decreases in expression of GPI-anchored proteins that do not bind zinc in *Z5Z7*-DKO cells using CD55, CD59, and ceruloplasmin (CP) as model GPI-anchored proteins. CP refers to the GPI anchor splicing variant form, which differs from the secretory form of CP (secCP) in the serum. Their expression substantially decreased in A549-*Z5Z7*-DKO cells compared with WT A549 cells detected by immunoblotting (Fig. 3*A*). The loss of CD55, CD59, and CP expression on the SK-*Z5Z7*-DKO cell surface was confirmed *via* immunofluorescence staining (Fig. 3*B*). Cell surface expression of CD55 and CD59 was restored by coexpression with ZNT5 or ZNT7 but not by zinc-transport incompetent ZNT5 or ZNT7 mutants (ZNT5_H451A_ and ZNT7_H70A_) (15, 25) when cultured even in the presence of 75 μM ZnSO_4_ (Fig. 3*C*). Cell surface expression of chimeric CD55-TM and CP-TM (GPI anchor attachment was substituted with the membrane-spanning polypeptide portion of ACE2 as described previously) was observed following transfection into SK-*Z5Z7*-DKO cells coexpressing ZNT5 or ZNT7 at the same level as WT cells (Fig. 3, *D* and *E*). Moreover, the expression of secCP was comparable between A549-*Z5Z7*-DKO and WT A549 cells (Fig. S1*C*). These results suggest that GPI-anchored proteins require ZNT5-6 or ZNT7 for cell surface expression.

### Impairment of endogenous GPI-anchored protein cell surface expression in *Z5Z7*-DKO cells

**Figure 4:**
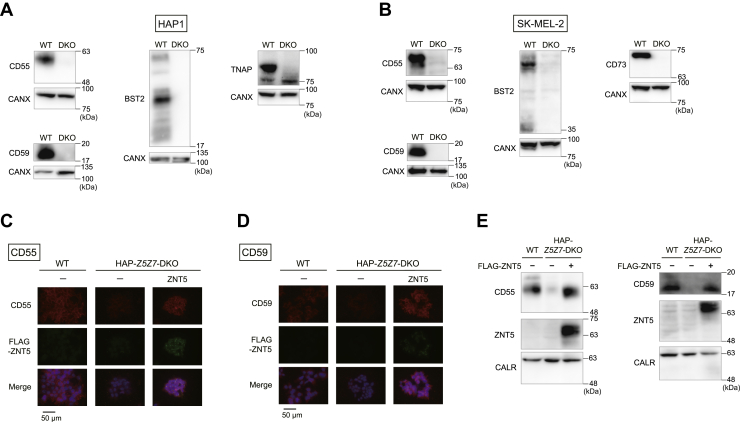
**Endogenous GPI-anchored proteins substantially decreased in *Z5Z7*-DKO cells.***A* and *B*, immunoblotting of endogenous CD55, CD59, BST2, and TNAP in WT HAP1 cells and HAP-*Z5Z7*-DKO cells (*A*) and WT SK-MEL-2 and SK-*Z5Z7*-DKO cells (*B*). *C* and *D*, CD55 and CD59 expression in HAP-*Z5Z7*-DKO cells following re-expression of FLAG-tagged ZNT5. Immunofluorescence staining was performed as in Figure 1. *E*, immunoblotting of CD55 and CD59 expression in HAP-*Z5Z7*-DKO cells following re-expression of FLAG-tagged ZNT5. In (*A*), (*B*), and (*E*), 20 μg of membrane protein prepared from the cells was used for immunoblotting analysis. Calnexin (CANX) or Calreticulin (CALR) is shown as a loading control. CNX was detected as the higher molecular weight band in the higher percentage gels. Each experiment was performed at least three times and representative results from independent experiments are shown. DKO, double KO; GPI, glycosylphosphatidylinositol; TNAP, tissue nonspecific ALP; ZNT, Zn transporter.

The change in expression of endogenous GPI-anchored proteins was examined using WT HAP1 and HAP-*Z5Z7*-DKO cells because TNAP GPI-anchored protein is substantially lower in HAP-*Z5Z7*-DKO cells than that in WT HAP1 cells (16). SWATH-MS analysis (26) of membrane fractions identified 2882 proteins and revealed the relative expression differences between HAP-*Z5Z7*-DKO cells and WT HAP1 cells (Table S1). Gene ontology term enrichment analysis of cellular components using the top 15 proteins revealed that the GPI anchor component was the most enriched protein type whose expression decreased (Table 2) and included BST2 (first), TNAP (fifth), glypican-4 (GPC4) (sixth), and CD55 (seventh, shown as DAF) as GPI anchor components (Table S1). Immunoblotting showed decreased expression of endogenous CD55, BST2, TNAP, and CD59 (unidentified in SWATH-MS analysis) in HAP-*Z5Z7*-DKO cells compared with WT HAP1 cells (Fig. 4*A*). Moreover, similar markedly decreased expression patterns were confirmed in WT SK-MEL-2 cells and SK-*Z5Z7*-DKO cells (CD73 was used instead of TNAP in SK-MEL-2 cells) (Fig. 4*B*). Immunofluorescence staining and immunoblotting confirmed the loss of CD55 and CD59 on the cell surface in HAP-*Z5Z7*-DKO cells (Fig. 4, *C* and *D*), which was restored by transfecting cells with plasmid expressing ZNT5 (Fig. 4, *C*–*E*). These results clearly showed that ZNT5-6 and ZNT7 play pivotal roles in the maturation and cell surface localization of endogenous GPI-anchored proteins.

**Table 2:** Enriched GO (cellular component) pathway showing decreased protein expression in HAP-*Z5Z7*-DKO cells compared with that in WT HAP1 cells using the top 15 proteins in SWATH-MS analysis

Term	Number within the top 15 proteins	Fold enrichment	Raw *p* value	FDR
Anchored component of membrane	4	47.62	1.06E-06	2.12E-03
Vacuolar lumen	3	35.92	6.92E-05	3.45E-02
Ficolin-1-rich granule	3	33.40	8.56E-05	3.42E-02
Secretory granule	5	12.02	2.68E-05	2.67E-02
Secretory vesicle	5	10.08	6.22E-05	4.14E-02
Extracellular region	8	3.81	1.13E-04	3.75E-02

Raw data of the SWATH-MS analysis is shown in Table S1. Fifteen proteins with the smallest DKO/WT ratios for protein expression levels underwent GO enrichment analysis (cellular component). Consequently, the six components are listed. The number within top the 15 proteins, fold enrichment, raw *p* value, and FDR were obtained *via* the original algorithm of the GO enrichment analysis website.

Abbreviations: FDR, false discovery rate; GO, Gene ontology.

### Examination of GPI-anchored protein expression in *Z5Z7*-DKO cells with impaired PIG protein function

**Figure 5:**
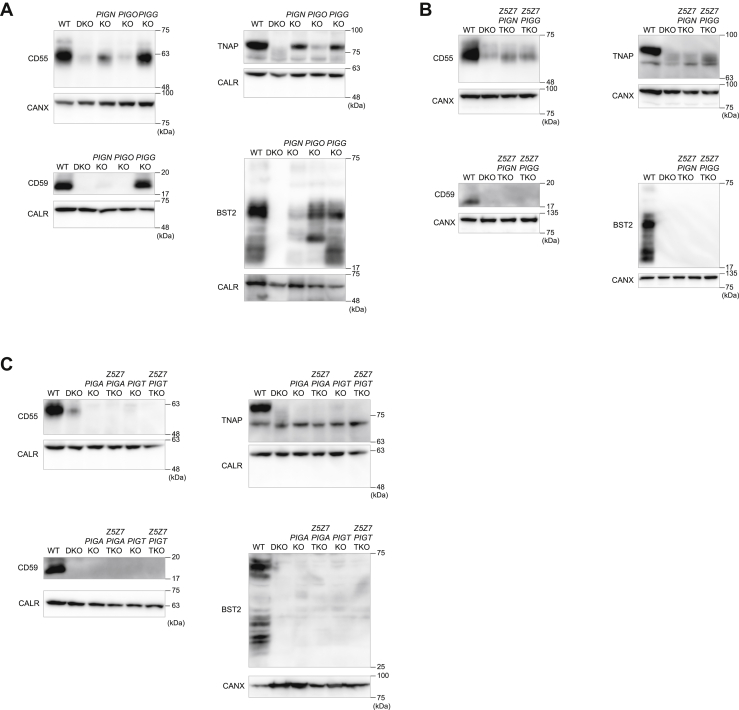
**GPI-anchored protein expression in *Z5Z7*-DKO cells with impaired PIG protein.***A*, CD55, CD59, TNAP, and BST2 expression in HAP-*PIGN*-KO, HAP-*PIGO*-KO, and HAP-*PIGG*-KO cells. *B*, CD55, CD59, TNAP, and BST2 expression in HAP-*Z5Z7PIGG*-TKO cells or HAP-*Z5Z7PIGN*-TKO cells. *C*, CD55, CD59, TNAP, and BST2 expression in HAP-*Z5Z7PIGA*-TKO cells, HAP-*Z5Z7PIGT*-TKO, HAP-*PIGA*-KO, and HAP-*PIGT*-KO cells. Twenty micrograms of membrane protein prepared from the cells was used for immunoblotting analysis. Calreticulin (CALR) or CANX is shown as a loading control. CANX was detected as the higher molecular weight band in the higher percentage gels. Each experiment was performed at least three times and representative results from independent experiments are shown. CANX, calnexin; DKO, HAP-*Z5Z7*-DKO cells; GPI, glycosylphosphatidylinositol; PIG, phosphatidylinositol glycan anchor biosynthesis; TNAP, tissue nonspecific ALP.

The GPI anchor is biosynthesized and assembled in sequential reactions by more than 20 PIG proteins (Fig. S2) (2, 3, 27). GPI-anchored protein expression is substantially decreased in cells deficient in most *PIG* genes (28). GPI ethanolamine phosphate transferases PIGN, PIGO, and PIGG are suggested to be zinc enzymes (29, 30, 31). We established HAP1 cells deficient in *PIGN*, *PIGO*, or *PIGG* (HAP-*PIGN*-KO, HAP-*PIGO*-KO, or HAP-*PIGG*-KO cells) and showed that the expression levels of BST2, CD55, CD59, and TNAP substantially decreased in HAP-*PIGO*-KO cells while the decrease in expression was minor in HAP-*PIGG*-KO cells and HAP-*PIGN*-KO cells (except for CD59) (Fig. 5*A*), which is consistent with a previous study (28). Meanwhile, BST2, CD55, CD59, and TNAP expression in HAP-*Z5Z7PIGG*-triple KO (TKO) cells and HAP-*Z5Z7PIGN*-TKO cells was almost identical to that in HAP-*Z5Z7*-DKO cells without any significant synthetic defects such as growth impairment (Fig. 5*B*). This suggested that the PIGG or PIGN defects merged with those of both ZNT5 and ZNT7 and that ZNT5-6 and ZNT7 supply zinc to these *PIG*s.

PIGA is an essential component of an enzyme complex involved in the first step of GPI anchor biosynthesis while PIGT functions in the final step of generating GPI-anchored proteins by transferring the biosynthesized GPI anchor to the C terminus (Fig. S2); thus, the impairment of both components results in loss of cell surface expression of GPI-anchored proteins (28, 32, 33). HAP-*PIGA*-KO and HAP-*PIGT*-KO cells showed substantially decreased expression of BST2, CD55, CD59, and TNAP (Fig. 5*C*). Moreover, substantial decreases were maintained in HAP-*Z5Z7PIGA*-TKO cells and HAP-*Z5Z7PIGT*-TKO cells, both of which grew in a similar manner to the respective KO cells and HAP-*Z5Z7*-DKO cells without any apparent synthetic defects. Thus, defects caused by the loss of PIGA or PIGT merged with those caused by deficiency of ZNT5-6 and ZNT7 functions in the GPI-anchored protein expression. These results strongly suggest that ZNT5-6 and ZNT7 contribute to GPI anchor biosynthesis conducted by PIG proteins in the ER, probably by supplying zinc to GPI ethanolamine phosphate transferases such as PIGN, PIGO, and PIGG.

### Disruption of both *Znt5* and *Znt7* genes causes touch-insensitivity in medaka fish

**Figure 6:**
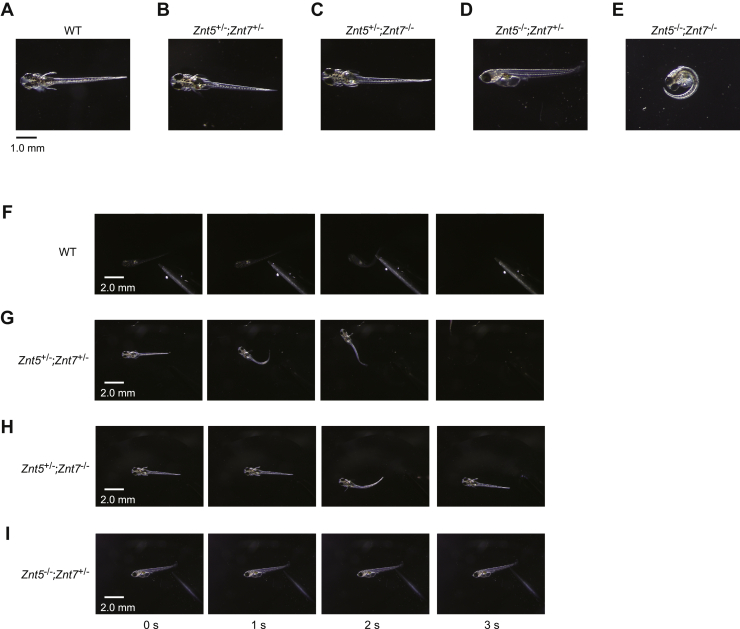
**Disruption of both *Znt5* and *Znt7* genes resulted in touch-insensitive phenotype in medaka fish (*Oryzias latipes*).***A*–*E*, representative lateral or dorsal, views of whole larvae of each genotype, 7 to 8 days postfertilization (dpf) in WT (*A*), *Znt5*^+/−^;*Znt7*^+/−^ (*B*), *Znt5*^+/−^;*Znt7*^−/−^ (*C*), *Znt5*^−/−^;*Znt7*^+/−^ (*D*), *Znt5*^−/−^;*Znt7*^−/−^ (*E*) medaka. Note that *Znt5*^−/−^;*Znt7*^+/−^, and *Znt5*^−/−^;*Znt7*^−/−^ larvae failed to show a dorsal photo because they did not move. *F*–*I*, mechanosensory stimulation induced swimming away in WT (*F*), *Znt5*^+/−^;*Znt7*^+/−^ (*G*), and *Znt5*^+/−^;*Znt7*^−/−^ (*H*), but not in *Znt5*^−/−^;*Znt7*^+/−^ (*I*) medaka. *Znt5*^−/−^;*Znt7*^+/−^ medaka did not respond to touch.

Zebrafish *Pigu* or *Pigk* mutants show touch-insensitive phenotypes due to impairment of Rohon–Beard sensory neuron excitability (20, 21). Medaka fish of homozygous *Znt5* KO (*Znt5*^−/−^) or homozygous *Znt7* KO (*Znt7*^−/−^), medaka heterozygous for *Znt5* and *Znt7* (*Znt5*^+/−^;*Znt7*^+/−^), medaka heterozygous for *Znt5* and homozygous for *Znt7* mutant (*Znt5*^+/−^;*Znt7*^−/−^), grew normally and survived into adulthood without any obvious morphological or developmental defects, as WT medaka (Fig. 6, *A*–*C* and Movies S1A–S3A). In contrast, medaka homozygous for *Znt5* and heterozygous for *Znt7* (*Znt*5^−/−^;*Znt7*^+/−^) showed a motionless side-lying phenotype after hatching (Fig. 6*D* and Movie S4*A*). Moreover, the double-homozygous mutant (*Znt5*^−/−^;*Znt7*^−/−^) also failed to move and showed a curled-up phenotype after manual hatching (Fig. 6*E*).

Each medaka was touched with a fine needle, except for the *Znt5*^−/−^;*Znt7*^−/−^ mutant. WT, *Znt5*^+/−^, *Znt7*^+/−^, and *Znt5*^+/−^;*Znt7*^−/−^ medaka sharply swam away from the stimuli (Fig. 6, *F*–*H* and Movies S1B–S3B) while *Znt*5^−/−^;*Znt7*^+/−^ did not respond and remained motionless (Fig. 6*I* and Movie S4*B*). These results are consistent with the touch-insensitive phenotype of the zebrafish *Pigu* or *Pigk* mutant (18, 19), although defects in both Znt5-6 and Znt7 functions showed more severe phenotypes. Taken together, ZNT5-6 and ZNT7 probably play essential roles in GPI-anchored protein biosynthesis *in vivo*, which is conserved in vertebrates.

## Discussion

ZNT5-6 and ZNT7 contribute to homeostatic maintenance of secretory pathway functions (10, 11, 12). In this study, TNAP, PLAP, and CD73 zinc ectoenzymes failed to be activated and had decreased expression in *Z5Z7*-DKO cells, which contrasts with aACE, gACE, ACE2, PLAP-TM(H), and PLAP-TM(G) (Figs. 1 and 2). Our results suggest that ZNT5-6 and ZNT7 activate ER-resident multi–membrane-spanning proteins PIGN, PIGO, and PIGG by supplying zinc. PIGN, PIGO, and PIGG are involved in different parts of GPI anchor biosynthesis in the ER (Fig. S2) (2, 3, 27, 28), while their loss contributes to the decrease in expression of GPI-anchored proteins. BST2, CD55, CD59, and TNAP expression was either substantially or minorly decreased in HAP-*PIGN*-KO, HAP-*PIGO*-KO, and HAP-*PIGG*-KO HAP1 cells (Fig. 5), while significant reductions in their expression were observed in the HAP1 cells deficient in *PIGN*, *PINO*, and *PIGG* genes (HAP-*PIGNPIGGPIGO*-TKO cells), which were comparable with those in HAP-*Z5Z7*-DKO cells (Fig. S3), further suggesting zinc supply to PIGN, PIGO, and PIGG by ZNT5-6 and ZNT7. It is unknown why these PIGs fail to be activated in *Z5Z7*-DKO cells because aACE, gACE, ACE2, PLAP-TM(H), and PLAP-TM(G) proteins with membrane-spanning polypeptide-anchored portions that are not GPI-anchored were expressed and activated in *Z5Z7*-DKO cells. Several possibilities exist to their loss of function. The active sites of PIGN, PIGO, and PIGG are homologous to ALPs (29), which are easily impaired in *Z5Z7*-DKO cells, as shown in reduced PLAP-TM(G) and PLAP-TM(H) activities (Fig. 2*A*) and secPLAP activity (Fig. S1*A*). Alternatively, we suggest that compared with ectoenzymes, intracellular zinc enzymes may be more easily impaired in *Z5Z7*-DKO cells because our recent study revealed that the activity of lysosomal-localized sphingomyelin phosphodiesterase 1 (SMPD1) (34, 35) is impaired in HAP-*Z5Z7*-DKO cells (36), although it is not a GPI-anchored zinc enzyme. Since GPI anchor biosynthesis and its transfer to proteins occurs in the ER (Fig. S2) (2, 3, 27, 28), our present study raises a fundamental question of how primarily Golgi apparatus localized ZNT5-6 and ZNT7 contribute to GPI-anchored protein maturation in the ER through zinc supply. It is suggested that either PIG proteins including PIGN, PIGO, and PIGG are retrogradely trafficked from the Golgi apparatus to the ER after zinc metalation in the Golgi apparatus or transient localization of ZNT5-6 and ZNT7 to the ER may contribute to supplying zinc to PIG proteins before their trafficking to the Golgi apparatus. We are currently investigating these mechanisms.

In *Z5Z7*-DKO cells, GPI-anchored protein expression was substantially decreased. Intracellular protein degradation may be a primary underlying cause for this observation, as reported for cells with loss-of-function mutations in most *PIG* genes (37). They may be degraded by the proteasome through ER-associated protein degradation (ERAD). GPI anchor proteins are known to be retrotranslocated for degradation by ERAD if GPI-anchored biosynthesis is prevented (38, 39) and misfolded GPI-anchored proteins accumulate in the presence of proteasome inhibitors (40, 41). Another possibility is that they may be degraded *via* the rapid ER stress–induced export degradation pathway. In this pathway, GPI-anchored proteins are eventually degraded in lysosomes (39, 42). Our assumption of these possibilities is derived from our previous results showing that TNAP expression was partially increased by the treatment of inhibitors of both proteasomal and lysosomal degradations (16, 25). However, degradation *via* the rapid ER stress–induced export pathway is unlikely because it is operative for misfolded GPI-anchored proteins after attachment of the GPI (3). In addition to intracellular degradation of GPI-anchored proteins, another possibility is that their pro-proteins lacking the GPI anchor attachment may be secreted into the extracellular space through escaping ERAD degradation in *Z5Z7*-DKO cells considering that a similar secretion is known to occur in loss-of-function mutations in some *PIG* genes (*PIGO* and two other *PIG* genes) (2, 37, 43). We detected very weak ALP activity in the cultured medium of HAP-*Z5Z7*-DKO cells but not in WT HAP1 cells (data not shown), which may support this possibility. We are also currently investigating the regulation of GPI-anchored protein expression in *Z5Z7*-DKO cells from these views.

In this study, *Znt*5^−/−^;*Znt7*^+/−^ medaka showed more severe phenotypes than zebrafish *Pigu* or *Pigk* mutants (20, 21). We speculate that this is because the defects caused by homozygous *Znt5* and heterozygous *Znt7* are broader and exacerbate other cellular functions, which may cause synthetic defects in GPI-anchored protein expression. Moreover, different phenotypes of *Znt*5^−/−^;*Znt7*^+/−^ medaka from that of *Znt5*^+/−^;*Znt7*^−/−^ indicate that ZNT5-6 and ZNT7 have different functions *in vivo*, although their contributions to zinc ectoenzyme activation are similar. Clarifying physiological differences between ZNT5-6 and ZNT7 is important because the *ZNT5/SLC30A5* mutation is responsible for perinatal lethal cardiomyopathy (44). A series of medaka mutants would be a useful tool to dissect the overlapping or specific functions of ZNT5-6 and ZNT7 *in vivo*.

GPI-anchored protein expression was the most significantly decreased protein type in HAP-*Z5Z7*-DKO cells compared with WT HAP1 cells according to Gene ontology term enrichment of the top 15 proteins in SWATH-MS data. Lysosomes were enriched when analyzing the top 500 proteins with reduced expression in HAP-*Z5Z7*-DKO cells compared with WT HAP1 cells, suggesting that ZNT5-6 and ZNT7 have broad biological functions in addition to GPI anchor biosynthesis. This is consistent with our recent finding that the lysosomal enzyme SMPD1 requires zinc mediated by ZNT5-6 and ZNT7 (36). Meanwhile, ER–Golgi transport was enriched when analyzing increased expression in HAP-*Z5Z7*-DKO cells compared with WT HAP1 cells, which may reflect the alleviation of GPI-anchored protein expression defects. Further experiments are required to determine whether these defects were directly affected by the loss of ZNT5-6 and ZNT7 functions or indirectly affected by the loss of GPI-anchored protein expression.

In conclusion, our results revealed a previously unappreciated insight into GPI-anchored protein expression, which requires zinc transport *via* ZNT5-6 and ZNT7. The results support previous literature showing that both complexes are intimately involved in protein quality control in the early secretory pathway. Considering that GPI-anchored proteins operate as enzymes, receptors, adhesion proteins, and complement regulatory proteins, the present results provide an important basis for understanding the physiological and molecular significance of zinc in biological processes that are dynamically mobilized by zinc transporters. Further studies are required to determine the molecular mechanism underlying GPI-anchored protein expression and dissect the critical role of ZNT5-6 and ZNT7 to improve our understanding of the crucial functions of zinc on protein quality control in the ER and the secretory pathway.

## Experimental procedures

### Cell culture

SK-MEL-2 cells containing the *Luc* gene (JCRB cell bank) were maintained at 37 °C in a humidified 5% CO_2_ incubator using RPMI1640 culture medium (FUJIFILM Wako Pure Chemical) containing 10% heat-inactivated fetal calf serum (Sigma–Aldrich), 100 U/ml penicillin, and 100 μl/ml streptomycin (Nacalai Tesque) in 10 cm dish (Thermo Fisher Scientific). Dulbecco’s modified Eagle’s medium (FUJIFILM Wako Pure Chemical) or Iscove’s modified Dulbecco’s medium (Nacalai Tesque) was used to maintain A549 or HAP1 cells as previously described (16, 25). Zinc-supplementation experiments involved adding the indicated concentrations of ZnSO_4_ (Nacalai Tesque) to the cell culture medium.

### Plasmid construction

Plasmid preparation for the expression of N-terminal HA-tagged or FLAG-tagged human ZNT5 (HA-ZNT5 or FLAG-ZNT5), C-terminal HA-tagged human ZNT7 (ZNT7-HA), and their zinc transport-incompetent mutants (HA-ZNT5_H451A_ or ZNT7_H70A_-HA) was performed as previously described (15, 16). Plasmids expressing *TNAP*, *PLAP*, *CD73*, s*ACE*, *ACE2*, g*ACE*, *CD55*, *CP*, and *CD59* were constructed by inserting full-length *TNAP*, *PLAP*, and *CD73* complementary DNAs (cDNAs) (17), s*ACE* cDNA (provided by Dr Eric Clauser, Collège de France) (45), *ACE2* cDNA (provided by Dr Shuetsu Fukushi, National Institute of Infectious Diseases) (46), or other cDNAs into pcDNA3 or IRES-GFP plasmids (provided by Dr Hirohide Saito, Kyoto University). IRES-GFP plasmid can discriminate cells expressing the subcloned cDNA using GFP fluorescence, when used in transfection studies. The g*ACE* cDNA was constructed by replacing the 5′ region of the s*ACE* gene coding the N-terminal domain of s*ACE* with the fragment of the 5′ region of the g*ACE* gene (containing the initiation codon ATG [A is referred to as +1] to a *Ppu*MI restriction site [+452]) (gBlock synthetic gene fragment, Integrated DNA Technologies). Sec*CP* expression plasmid was purchased from DNAFORM, which was fused in frame with a cDNA fragment encoding its GPI anchor to generate its GPI-anchored form (CP) as described elsewhere (47). *CD55* and *CD59* cDNAs were amplified by PCR using human neural cDNA (DV Biologics) or human pancreatic islet cDNA (Cosmo Bio Co, Ltd) as a template. ACE-GPI and ACE2-GPI fusion plasmids were constructed by replacing the 3′ regions of *ACE* and *ACE2* encoding the C-terminal transmembrane region with the 3′ region of the *PLAP* gene encoding the GPI anchor attached site using two-step PCR using KOD-PLUS Taq polymerase (Toyobo). The cycle conditions were denatured at 95 °C for 5 min and amplified for 35 reaction cycles with denaturation at 95 °C for 10 s, annealing at 60 °C for 30 s, extension at 68 °C for 2 min per cycle, and a final extension step at 68 °C for 10 min. CP-TM and CD55-TM fusion plasmids were constructed by replacing the 3′ regions of *CP* or *CD55* encoding the portions of their GPI attachment with that of the membrane-spanning polypeptide portion of *ACE2*. The secretory form of ACE (secACE-HA) was constructed by replacing the 3′ region encoding the C-terminal transmembrane region with the fragment encoding the HA tag. The secretory form of PLAP (secPLAP-HA) has been previously described (16). PLAP-TM(H) or PLAP-TM(G) expression plasmids were constructed by inserting cDNA (provided by Dr Deborah Brown, Stony Brook University) (24) into the pcDNA3 plasmid. Plasmids expressing β-galactosidase (pβactβgal) were described elsewhere (48).

### Disruption of *ZNT5* and *ZNT7* or *PIGN*, *PIGO*, *PIGG*, *PIGA*, and *PIGT* genes

*ZNT5* and *ZNT7* genes were simultaneously disrupted by CRISPR/Cas9–mediated genome editing using single guide RNA expression plasmids as previously described (16). The constructed plasmids (4 μg) and one-tenth quantity of pA-puro vector (25) containing the puromycin resistance gene for A549 cells or pcDNA3 containing the neomycin resistance gene for SK-MEL-2 cells were cotransfected into 80% confluent cells using 4 μl Lipofectamine 2000 (Invitrogen). After culturing for 1 day, the cells were transferred to a 10 cm cell culture dish and cultured in the presence of 4 μg/ml puromycin (InvivoGen) for A549 cells or 2 mg/ml G418 (Nacalai Tesque) for SK-MEL-2 cells, respectively, to establish stable clones. HAP-*Z5Z7*-DKO cells were established in a previous study (16). HAP1 cells deficient in *PIGN*, *PIGO*, *PIGG*, *PIGA*, or *PIGT* genes were established in the same manner as previously described (16). To generate HAP-DKO or HAP-TKO cells, the cells were cultured in the presence of 20 μg/ml blasticidin S (InvivoGen), 0.75 μg/ml puromycin or 1.5 mg/ml G418. Oligonucleotides used for the generation of single guide RNA expression plasmids are listed in Table S2. Gene-edited cells were confirmed by sequencing the genomic DNA amplified PCR fragments using the primers listed in Table S3.

### Transient and stable transfection

Cells were seeded into 12-well plates (1.0 × 10^5^ cells/well for A549 and 1.2 × 10^5^ cells/well for SK-MEL-2) and cultured for 24 h. A549 cells were transfected with 1 μg of empty pcDNA3 or pcDNA3 harboring each cDNA with 0.2 μg of pβactβgal plasmid (for transfection efficiency normalization) in Opti-MEM reduced serum media (Thermo Fisher Scientific) using 1.5 μl Lipofectamine 2000. The same strategy was used for transient transfection of secretory enzymes except that 0.2 μg of pCMV-*Gaussia* Luc plasmid (Thermo Fisher Scientific) was used to express secretory *Gaussia* luciferase instead of the pβactβgal plasmid. SK-MEL-2 cells were transfected with 0.5 μg of empty IRES-GFP plasmid or IRES-GFP plasmid harboring each cDNA. The transfection medium was replaced after 8 h and 4 h for A549 cells and SK-MEL-2 cells, respectively, with the corresponding culture medium, and cells were cultured for an additional 16 h and 24 h for A549 cells and SK-MEL-2 cells, respectively, prior to the experiments. Stable transfection in HAP1 cells (seeded at 5 × 10^5^ cells/ml in a 6 cm dish) used 4 μg of the respective expression plasmids in Opti-MEM using 4 μl Lipofectamine 2000. Transfected cells were transferred to a 10 cm cell culture dish and cultured in the presence of 0.75 μg/ml puromycin as previously described (16). More than three stable independent clones were established per transfectant in all the experiments.

### Immunoblotting

Immunoblotting was performed as previously described (16, 17), except that cell lysates or membrane fractions were analyzed using nonreducing (-DTT) SDS-PAGE for detecting CD55. Blotted polyvinylidene fluoride membranes (Millipore) were blocked for 1 h with 5% skim milk and 0.1% Tween 20 in PBS (137 mM NaCl, 2.68 mM KCl, 1.47 mM KH_2_PO_4_, 8.1 mM NaH_2_PO_4_, pH 7.4) and incubated with primary antibodies (Table S4) diluted in blocking solution. The blot was washed in 0.1% Tween 20 in PBS, followed by incubation for 1 h with 1:3000 dilutions of horseradish peroxidase–conjugated antimouse, anti-rabbit, or antirat secondary antibodies (NA931, NA934, or NA935; Cytiva). Immunoreactive bands were detected using Immobilon Western chemiluminescent horseradish peroxidase substrate (Millipore) or SuperSignal West Femto maximum sensitivity substrate (Pierce; Thermo Fisher Scientific) according to the manufacturer’s instructions. Chemiluminescence images were obtained using ImageQuant LAS 500 (Cytiva).

### Immunofluorescence staining

Immunostaining was mainly performed in SK-MEL-2 cells because little nonspecific staining was observed when the primary antibodies (Table S4) were applied to the cells. The cells were cultured on coverslips coated with 0.01% poly-L-lysine (Sigma–Aldrich) and fixed with 10% formaldehyde neutral buffer solution (Nacalai Tesque). Meanwhile immunodetection of CD55 and CD59 was performed in HAP1 cells fixed with methanol. Cells were incubated with primary antibodies followed by secondary antibodies (Alexa 594–conjugated goat anti-mouse immunoglobulin G [IgG], Alexa 594–conjugated goat anti-rat IgG, Alexa 488–conjugated donkey anti-rabbit IgG, or Alexa 488–conjugated goat anti-rabbit IgG) and tertiary antibodies (Alexa 594–conjugated donkey anti-goat IgG or Alexa 594–conjugated rabbit anti-goat IgG) (Thermo Fisher Scientific) without permeabilization. Alexa 488–conjugated donkey anti-rabbit IgG and Alexa 594–conjugated donkey anti-goat IgG (Abcam) were also used as secondary and tertiary antibodies. The antibodies were applied for 1 h at room temperature or at 4 °C overnight, and 5 μg/ml 4,6-diamino-2-phenylindole (Thermo Fisher Scientific) was added during the second or third antibody staining to label nuclei. After three PBS washes, the coverslips were mounted onto the glass slides using SlowFade Diamond Antifade Mountant reagent (Thermo Fisher Scientific). The stained cells were examined using an Olympus FSX100 fluorescence microscope. Identical exposure settings and times were used for the corresponding images in each Figure.

### Measurement of PLAP and TNAP activity

Total cellular lysates prepared from transfected cells lysed in ALP lysis buffer (10 mM Tris–HCl, 0.5 mM MgCl_2_, 0.1% Triton X-100，pH 7.5) were used to measure TNAP or PLAP activity as previously described (15). Lysates were preincubated for 10 min at room temperature (RT), followed by the addition of 100 μl substrate solution (2 mg/ml disodium *p*-nitrophenylphosphate hexahydrate or *p*NPP; Wako Pure Chemicals, in 1 M diethanolamine buffer pH 9.8 containing 0.5 mM MgCl_2_), and the released *p*-nitrophenol product was quantified by measuring the absorbance at 405 nm using a Synergy H1 Hybrid multimode microplate reader (BioTek). Calf intestinal ALP (Promega) was used to generate a standard curve. The samples were incubated at 65 °C for 30 min to measure PLAP activity to discriminate between other activities.

### Measurement of CD73 activity

CD73 activity was evaluated using the malachite green assay as previously described (17). Briefly, 5 μg of total cell lysate prepared from transfected cells in 200 μl of CD73 lysis buffer (10 mM Tris–HCl pH 7.5, 0.1% Nonidet P-40) was incubated at 37 °C for 10 min, followed by incubation with 2 mM AMP (Oriental Yeast Co, Ltd) at 37 °C for 30 min. Fifty microliter of the reaction solution was mixed with 100 μl of Biomol Green (Enzo Life Sciences) and incubated for 10 min at RT. The amount of inorganic phosphate released from AMP was quantified by measuring the absorbance at 595 nm using a Synergy H1 Hybrid multimode microplate reader. A standard curve was generated using 0 to 200 μM Na_2_HPO_4_ solutions dissolved in CD73 lysis buffer.

### Measurement of ACE and ACE2 activity

ACE and ACE2 activity was measured using a previous method (49, 50). Briefly, 3 μg (5 μl) of total cellular lysate prepared from transfected cells was incubated at 37 °C for 10 min, followed by the addition of 45 μl 20 μM Nma-Phe-His-Lys(Dnp) for ACE and 20 μM Nma-His-Pro-Lys(Dnp) for ACE2 (Peptide Institute) in 0.1 M Hepes pH 7.5, 0.3 M NaCl, 0.02% Tween 20 (or 0.01% Triton X-100 for ACE2) and incubated at 37 °C for 30 min. The increase in fluorescence intensity (340 nm excitation, 440 nm emission) was measured using a Synergy H1 Hybrid multimode microplate reader. Recombinant ACE or ACE2 (10 μg) (BioLegend) was used to generate standard curves.

### Measurement of β-gal activity

Total cellular lysate prepared from transfected cells was incubated at 37 °C for 10 min, and 120 μl of buffer A (100 mM NaH_2_PO_4_, 10 mM KCl, 1 mM MgSO_4_, 50 mM β-mercaptoethanol, pH 7.5) was added to each well. The plate was incubated at 37 °C for 5 min followed by the addition of 50 μl 0.1 mM *o*-nitrophenyl *β*-D-galactopyranoside (FUJIFILM Wako Pure Chemical) and incubation at 37 °C for 60 min. The released *o*-nitrophenol was quantified by measuring the absorbance at 405 nm. *β*-D-galactosidase purified from *Escherichia coli* (FUJIFILM Wako Pure Chemical) was used to generate a standard curve.

### SWATH-MS analysis

Isolation of the membrane fraction was described elsewhere (51) with minor modifications. HAP1 cells cultured on 4 × 10 cm dishes were collected, washed with cold PBS, and homogenized in homogenization buffer (225 mM mannitol, 75 mM sucrose, 30 mM Tris–HCl, and 100 μM EGTA, pH 7.4) using a Dounce homogenizer. The homogenate was centrifuged twice at 600*g* for 5 min at 4 °C to eliminate the pellet containing nuclei, 7000*g* for 10 min at 4 °C to remove mitochondria, and the supernatant containing membrane fractions consisting of microsomes, plasma membranes, and lysosomes was centrifuged at 100,000*g* for 60 min at 4 °C. The supernatant was discarded, while the purified membrane fractions were stored at −80 °C.

SWATH-MS analysis was performed as described previously (52). Fifty micrograms of membrane protein fractions prepared from WT HAP1 cells and HAP-*Z5Z7*-DKO cells were solubilized in denaturing buffer (7 M guanidium hydrochloride, 0.5 M Tris–HCl (pH 8.5), and 10 mM EDTA). The solubilized proteins were reduced by DTT for 1 h at 25 °C, and, subsequently, S-carboxymethylated with iodoacetamide for 1 h at 25 °C. The alkylated proteins were precipitated with methanol–chloroform–water mixture. The precipitates were solubilized in 6 M urea in 0.1 M Tris–HCl (pH 8.5) and diluted fivefold with 0.1 M Tris–HCl (pH 8.5) containing 0.05% ProteaseMax surfactant (Promega). The dilutions were reacted with lysyl endopeptidase (Lys-C; Wako Pure Chemical) at an enzyme/substrate ratio of 1:100 for 3 h at 30 °C. Subsequently, Lys-C digested proteins were treated with TPCK-treated trypsin (Promega) at an enzyme/substrate ratio of 1:100 for 16 h at 37 °C. After C18 clean up, the peptide samples were injected into a NanoLC Ultra system (Eksigent Technologies) coupled with an electrospray-ionization TripleTOF 5600 mass spectrometer (SCIEX), which was set up for a single direct injection and analyzed by SWATH-MS acquisition. The peptides were directly loaded onto a self-packed C18 analytical column, prepared by packing ProntoSIL 200-3-C18 AQ beads (3 μm, 120 Å; BISCHOFF Chromatography) in a PicoFrit tip (ID 75 μm, PF360-75-10-N5; New Objective) of 20 cm length. After sample loading, the peptides were separated and eluted with a linear gradient; 98% A: 2% B to 65% A: 35% B (0–120 min), increase to 0% A: 100% B (120–121 min), maintained at 0% A: 100% B (121–125 min), reduced to 98% A: 2% B (125–126 min), and then maintained at 98% A: 2% B (126–155 min). Mobile phase A composition was 0.1% formic acid in water, and mobile phase B contained 0.1% formic acid in acetonitrile. The flow rate was 300 nl/min. The eluted peptides were positively ionized and measured in the SWATH mode. The measurement parameters are described as follows: SWATH window, 64 variable windows from 400 m/z to 1200 m/z; product ion scan range, 50 to 2000 m/z; declustering potential, 100; rolling collision energy value, 0.0625 × [m/z of each SWATH window] − 3.5; collision energy spread, 15; accumulation time, 0.05 s for each SWATH window.

Spectral alignment and data extraction from SWATH data were performed with the SWATH Processing Micro App in Peakview version 2.2 (SCIEX) using two spectral libraries, an in-house spectral library and a publicly available pan human library (PHL), for increasing the identification number of expressed proteins. The parameters for peak data extraction by Peakview were described as follows: number of peptide per protein, 999; number of transitions per peptide, 6; peptide confidence threshold, 99%; false discovery rate threshold, 1.0%; XIC extraction window, ±4.0 min; XIC width (ppm), 50. The detail of data analysis is described in Fig. S4. This data analysis improves the accuracy of protein identification and quantification by selecting only those peptides that meet the *in silico* peptide selection criteria (53). The peptide selection criteria are stringent, which increases the reliability of quantification, but reduces the number of peptides used to quantify individual proteins; as shown in Table S1, there are proteins that are quantified with a single peptide. The four GPI anchor components (BST2, TNAP, GPC4, and CD55), which are the most important in this study, were also analyzed by another data analysis procedure (Fig. S5). Quantification by two or more peptides ensures reliability (Table S5). In addition to the quantification results at the protein level, Table S5 also shows the peptide sequences used in the quantification analysis for individual proteins.

### Plasmid construction and microinjection to generate KO medaka

CRISPR/Cas9 was used as previously described (54) to edit *Znt5* and *Znt7* genes in medaka. Briefly, KO plasmid p2BaitD-Znt5-Cry-mCherry (Fig. S6*A*) was designed such that the *mCherry* gene driven by the crystalline promoter was inserted into the 5′ region encoding the putative transmembranous zinc-binding HDHD motif (9) in exon 11 by homologous recombination to generate *Znt5*-deficient medaka. Meanwhile, KO plasmid p2BaitD-Znt7-Cry-EGFP (Fig. S6*B*) was designed such that the *EGFP* gene driven by the crystalline promoter was inserted into the sequence encoding the putative zinc-binding HDHD motif in exon 3 by homologous recombination to generate *Znt7*-deficient medaka. The plasmids were generated by PCR amplification and ligation using primers listed in Table S6. Specifically, the left homology arm (LHA), right homology arm (RHA) (Table S7), and crystalline-promoter-mCherry-SV40polyAsignal (Cry-mCherry) or crystalline-promoter-EGFP-SV40polyAsignal (Cry-EGFP) were separately amplified by PCR. Then, the three fragments were combined using PCR to generate the LHA-Cry-mCherry-RHA and LHA-Cry-EGFP-RHA fragment, respectively. The fragment was digested with *Xho*I and *Spe*I and ligated into plasmid “p2BaitD-p4hb_500 bp-LUC-EGFP” (54) to generate p2BaitD-Znt5-Cry-mCherry and p2BaitD-Znt7-Cry-EGFP.

The mixture containing crispr RNA for *Znt5* or *Znt7* (50 ng/μl) (FASMAC), crispr RNA for BaitD (50 ng/μl) (FASMAC), tracrRNA (200 ng/μl) (FASMAC), Cas9 nickase (D10A) protein (1000 ng/μl) (Integrated DNA Technologies), and the KO plasmid (2.5 ng/μl) was injected into the medaka egg cytoplasm before first cleavage occurred as previously described (55).

### Establishment and care of *Znt*-deficient medaka

All medaka strains including WT were maintained in an aquarium with recirculating water in a 14/10 h light/dark cycle at 26 °C, which meets the Regulation for Animal Experiments in Kyoto University approved by the Animal Research-Animal Care Committee of Kyoto University (R3-45 and Lif-K21023).

The injected G0 embryos were cultured and bred into adults. The G0 fish were mated with WT counterparts, and F1 individuals with red fluorescence for Znt5 or green fluorescence for Znt7 in the eyes were selected. The F1 individual was mated with the WT counterpart, and the resulting heterozygous F2 individuals deficient in Znt5 or Znt7 were selected. In addition, continuous mating of F1 individuals with red or green eye fluorescence with their descendants produced individuals with various genotype combinations (*Znt5*^+/−^:*Znt7*^+/−^, *Znt5*^+/−^:*Znt7*^−/−^, *Znt5*^−/−^:*Znt7*^+/−^, *Znt5*^−/−^:*Znt7*^−/−^). Each genotype was confirmed *via* PCR using KOD-FX (Toyobo) in the PCR cycling conditions: denaturation for 2 min followed by 30 cycles at 98 °C for 10 s, annealing at 55 °C for 10 s and extension at 68 °C for 30 s for Znt5 and Znt7 alleles. Primers used for PCR amplification and DNA sequencing are listed in Table S8.

### Statistical analyses

All data are expressed as the mean ± SD of triplicate experiments. Statistical significance was determined by two-tailed Student’s *t* test at *p* < 0.01 (∗∗).

## Data availability

All data generated or analyzed during this study are included in this published article and its supporting information file or are available from the corresponding author (Taiho Kambe, Kyoto University, E-mail: kambe.taiho.7z@kyoto-u.ac.jp) upon reasonable request. Full-length immunoblots corresponding to images in the main text and supplementary figures are shown in Figs. S7–S13.

The mass spectrometry proteomics data have been deposited to the ProteomeXchange Consortium *via* the PRIDE (56) partner repository with the dataset identifier PXD032172.

## Supporting information

This article contains supporting information (3, 27).

## Conflict of interest

The authors declare that they have no conflicts of interest with the contents of this article.

## Supporting information

Movie S1A

Movie S1B

Movie S2A

Movie S2B

Movie S3A

Movie S3B

Movie S4A

Movie S4B
