# Supplementary material for: Zinc transport via ZNT5-6 and ZNT7 is critical for cell surface glycosylphosphatidylinositol-anchored protein expression
Source: J Biol Chem. 2022 May 4;298(6):102011. doi: 10.1016/j.jbc.2022.102011 (PMC9168625; doi:10.1016/j.jbc.2022.102011)
Supplement: Supplemental Figures S1–S13, Tables S2–S4 and S6–S8 [file mmc9.docx]

**Zinc transport *via* ZNT5-6 and ZNT7 is critical for cell surface glycosylphosphatidylinositol-anchored protein expression**

**Takumi Wagatsuma^1,^*, Keiko Shimotsuma^1,^*, Akiko Sogo^2^, Risa Sato^3^, Naoya Kubo^1^,　Sachiko Ueda^1^, Yasuo Uchida^3^, Masato Kinoshita^2^ and Taiho Kambe^1^**

*These authors contributed equally to this work.

^1^Division of Integrated Life Science, Graduate School of Biostudies, Kyoto University, Kyoto 606-8502, Japan

^2^Division of Applied Biosciences, Graduate School of Agriculture, Kyoto University, Kyoto 606-8502, Japan

^3^Division of Membrane Transport and Drug Targeting, Graduate School of Pharmaceutical Sciences, Tohoku University, 980-8578 Sendai, Japan.

**TABLE OF CONTENTS**

**Figure S1. Secretory forms of zinc ectoenzymes in the spent medium of transfected A549-*Z5Z7*-DKO cells.**

**Figure S2. PIG proteins involved in GPI anchor biosynthesis.**

**Figure S3. GPI-anchored protein expression in HAP-*PIGNPIGOPIGG*-TKO cells.**

**Figure S4. Data analysis procedure for the SWATH-MS experiment.**

**Figure S5. Data analysis procedure associated with the quantification of GPI anchor components (BST2, TNAP, GPC4 and CD55) using more peptides.**

**Figure S6. Schematic diagram of p2BaitD-Znt5-Cry-mCherry and p2BaitD-Znt7-Cry-EGFP used for medaka gene editing.**

**Figure S7. Full-length immunoblot images used in Figure 1.**

**Figure S8. Full-length immunoblot images used in Figure 2.**

**Figure S9. Full-length immunoblot images used in Figure 3.**

**Figure S10. Full-length immunoblot images used in Figure 4.**

**Figure S11. Full-length immunoblot images used in Figure 5.**

**Figure S12. Full-length immunoblot images used in Figure S1.**

**Figure S13. Full-length immunoblot images used in Figure S3.**

**Movie S1. Movement of WT medaka (A), when touched with a fine needle (B).**

**Movie S2. Movement of *Znt*5^+/-^;*Znt7*^+/-^ medaka (A), when touched with a fine needle (B).**

**Movie S3. Movement of *Znt5*^+/-^;*Znt7*^-/-^ medaka (A), when touched with a fine needle (B).**

**Movie S4. Motionless of *Znt5*^-/-^;*Znt7*^+/-^ medaka (A), when touched with a fine needle (B).**

**Table S1. Reliable data of the relative protein abundance between HAP-*Z5Z7*-DKO cells and WT HAP1 cells (refer to the Excel file).**

**Table S2. Oligonucleotides used for the generation of sgRNA expression plasmids.**

**Table S3. Primers used for genomic PCR to confirm gene editing in KO cells.**

**Table S4. Primary antibodies used in immunoblot (IB) analysis and immunofluorescence (IF) staining.**

**Table S5. Reliable data of four GPI anchor components (BST2, TNAP, GPC4 and CD55) using more peptides (refer to the Excel file).**

**Table S6. Primers used to generate *Znt5* and *Znt7*-KO medaka.**

**Table S7. Sequence information used for editing *Znt5* and *Znt7*-KO medaka.**

**Table S8. Primers used for PCR amplification and DNA sequencing to confirm the genotypes of medaka.**

**Figure S1. Secretory forms of zinc ectoenzymes in the spent medium of transfected A549-*Z5Z7*-DKO cells.** A-B. PLAP activity of secPLAP-HA (*A*) and secACE-HA (*B*) in the spent medium of transfected cells (*upper* panels). A-B (*lower* panels) and C. Immunoblot detection of secPLAP-HA (*A*), secACE-HA (*B*), and secCP (*C*) in the spent medium. Transient expression of *Gaussia* luciferase (GLuc) was used as the internal control together with expression of plasmids for each cDNA. Either 4 or 20 μL of the spent medium was used for the activity assays (*upper* panels of *A* and *B*) or immunoblot analysis (*lower* panels). DKO refers to double knockout. Each experiment was performed at least three times and representative results from independent experiments are shown.

**Figure S2. PIG proteins involved in GPI anchor biosynthesis.** More than 20 PIG proteins involved in GPI anchor biosynthesis are shown in sequential reactions. Step 3 is the flipping step from the cytosol to luminal side of the ER while step 5 is the lipid remodeling step (3, 27). Red colored PIG proteins were disrupted in HAP1 cells in this study. DPM2 is a subunit of GPI N-acetylglucosaminyl transferase comprising 7 proteins listed in Step 1, while GPAA1 is a subunit of GPI transamidase comprising 5 proteins listed in Step 12.

**Figure S3. GPI-anchored protein expression in HAP-*PIGNPIGOPIGG*-TKO cells.** CD55, CD59, TNAP, and BST2 expression in HAP-*PIGNPIGOPIGG*-TKO cells, HAP-*PIGN*-KO, HAP-*PIGO*-KO, and HAP-*PIGG*-KO cells. Twenty μg of membrane proteins prepared from cells were subjected to immunoblot analysis. Calnexin (CANX) is shown as a loading control. DKO, HAP-*Z5Z7*-DKO cells. Each experiment was performed at least three times and representative results from independent experiments are shown.


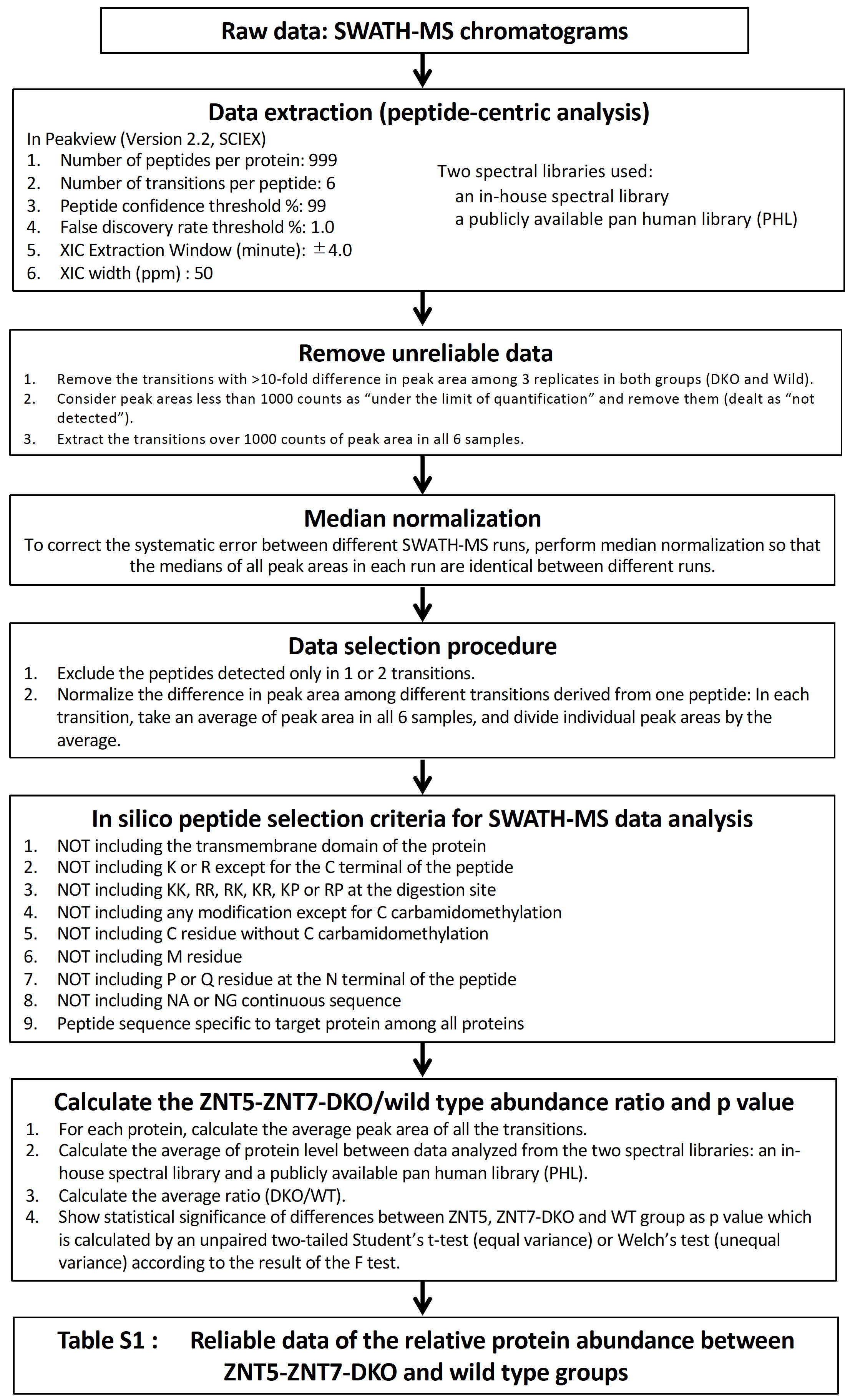


**Figure S4. Data analysis procedure for the SWATH-MS experiment.**


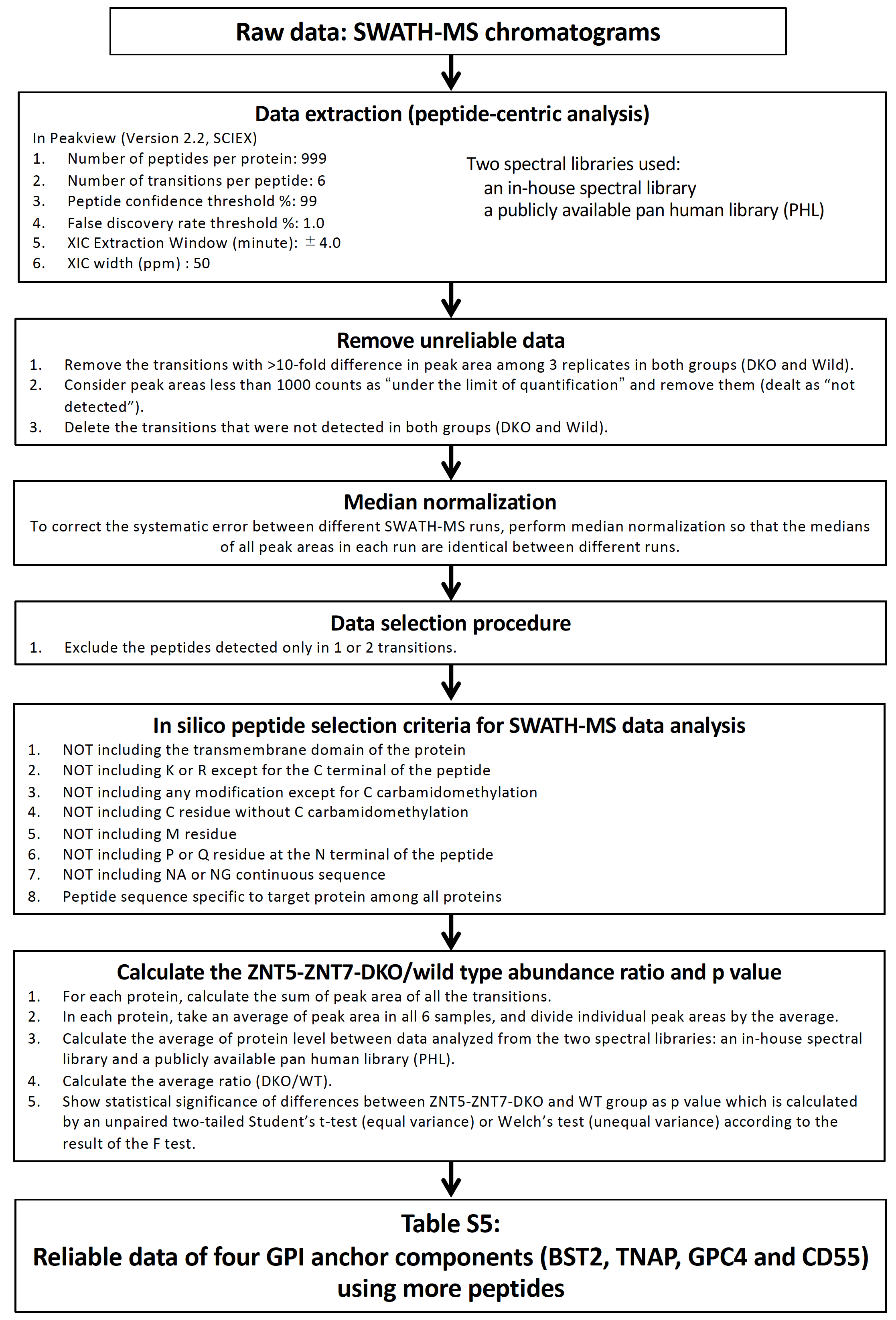


**Figure S5. Data analysis procedure associated with the quantification of GPI anchor components (BST2, TNAP, GPC4 and CD55) using more peptides.**

**
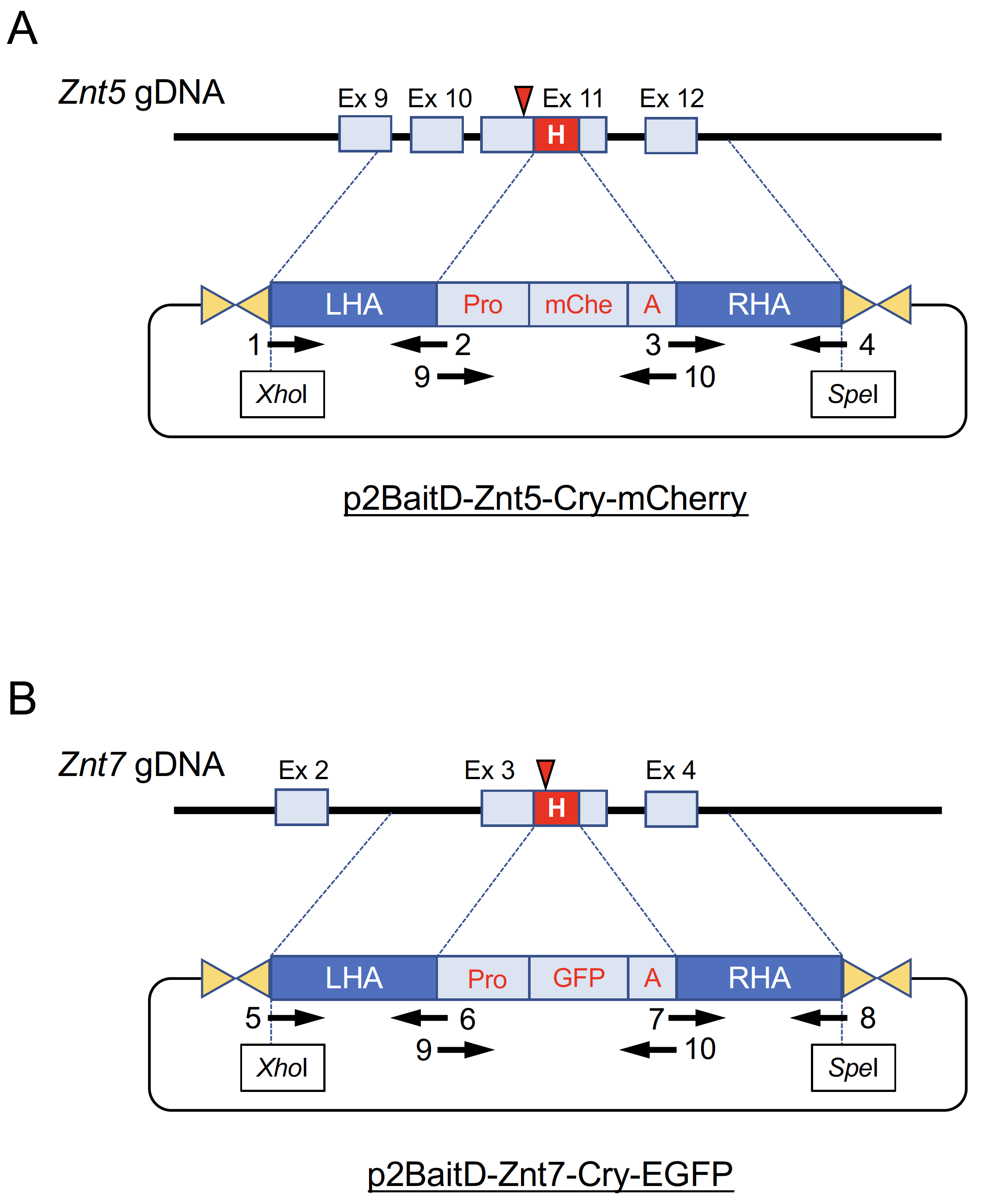
**

**Figure S6. Schematic diagram of p2BaitD-Znt5-Cry-mCherry and p2BaitD-Znt7-Cry-EGFP used for medaka gene editing.** A. p2BaitD-Znt5-Cry-mCherry. B. p2BaitD-Znt7-Cry-EGFP. “H” in exon 11 and 3 represents the HDHD motif in panel *A* and *B*, respectively. The arrowhead shows the target position for crRNA and numbered arrows are the PCR primers (Supplementary Table 5). Ex: exon; LHA: left homology arm; RHA: right homology arm; Pro: Crystalline promoter; mChe: *mCherry* gene; GFP: *EGFP* gene; A: SV40 polyA signal.


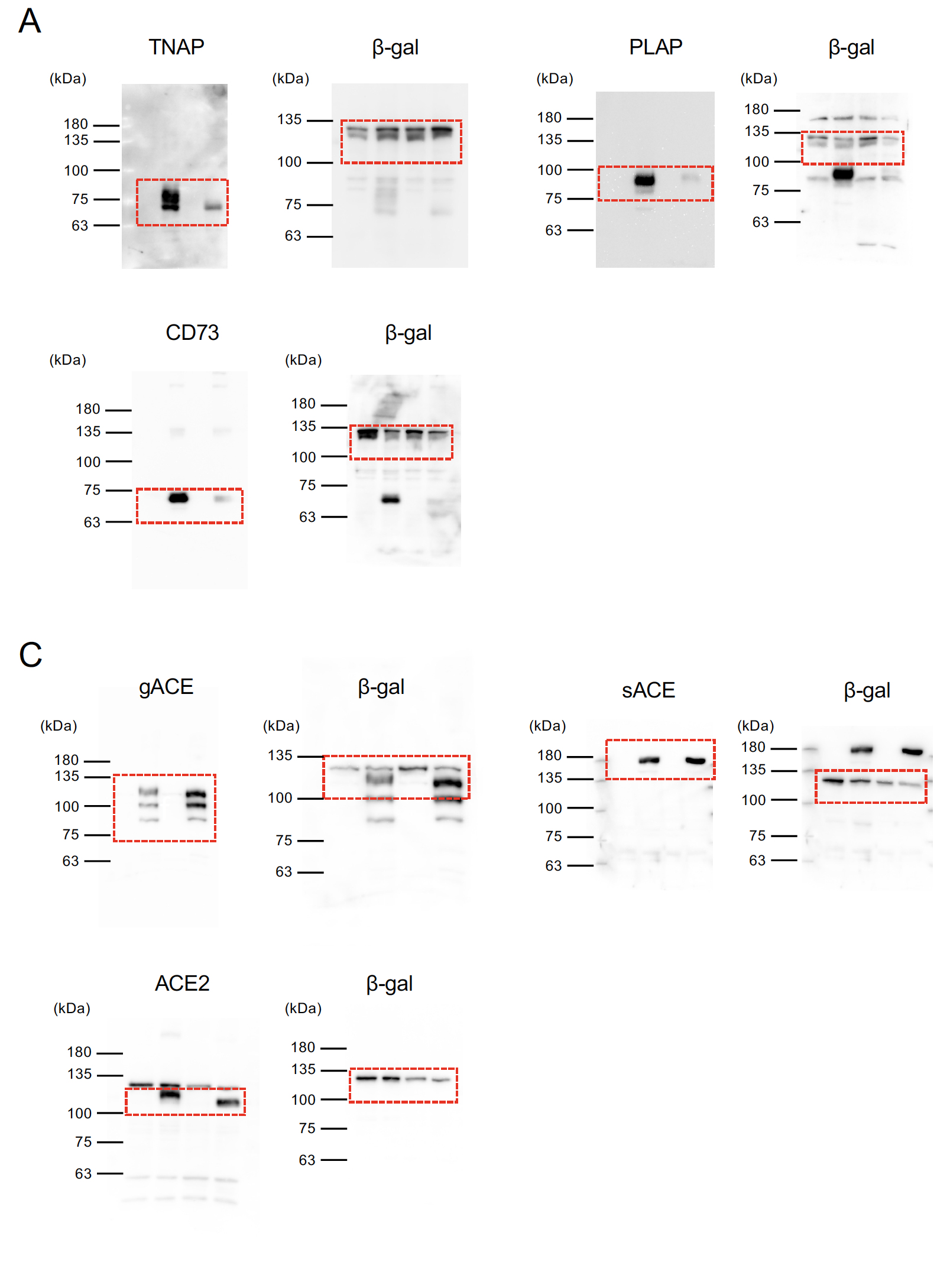


**Figure S7. Full-length immunoblot images used in Figure 1.** The panel used is boxed. The same blot was used sequentially (after stripping) for detection in each composite figure. The molecular weights of the marker proteins are indicated on the left of the immunoblot images.


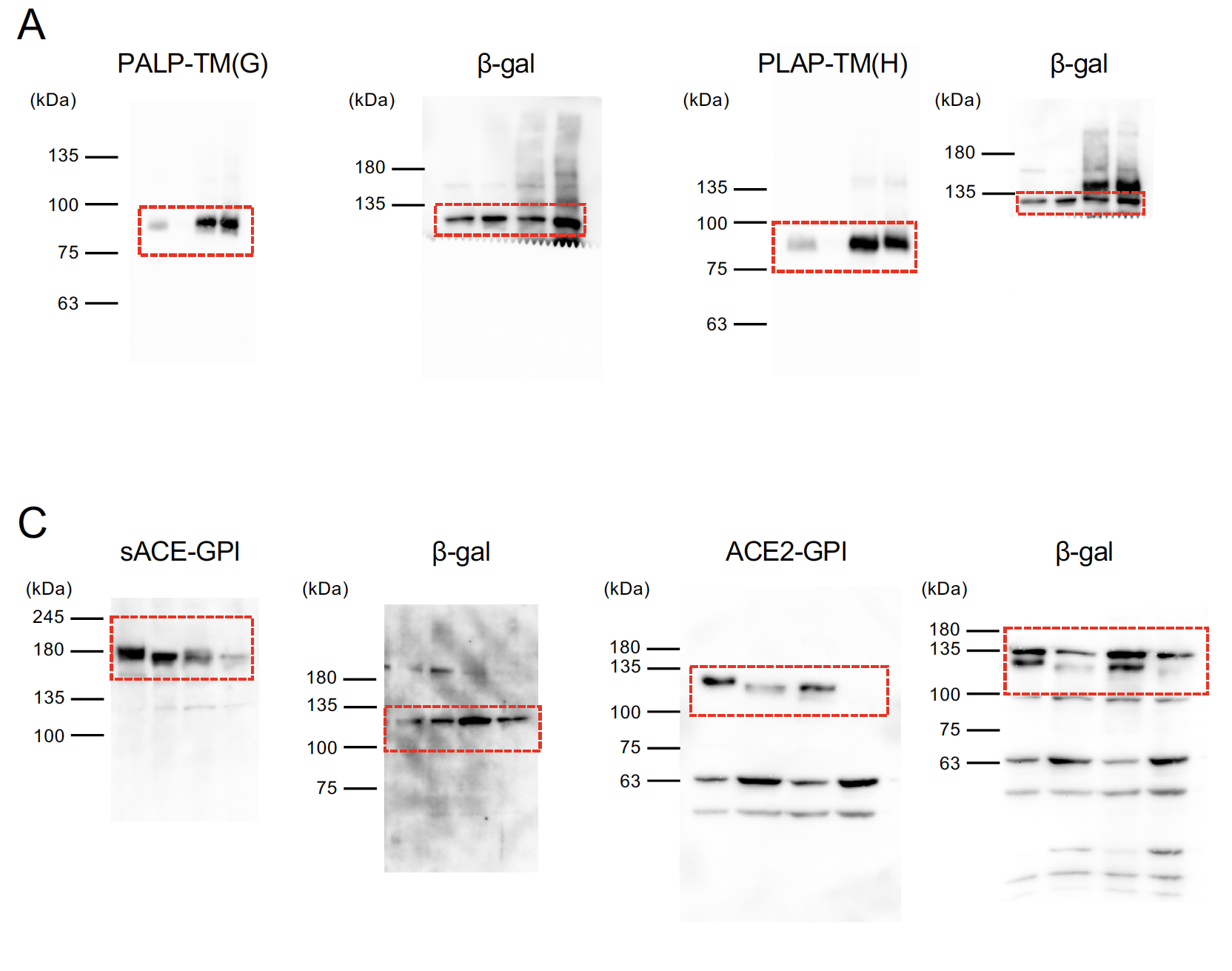


**Figure S8. Full-length immunoblot images used in Figure 2.** The panel used is boxed. The same blot was used sequentially (after stripping) for detection in each composite figure. The molecular weights of the marker proteins are indicated on the left of the immunoblot images.

**
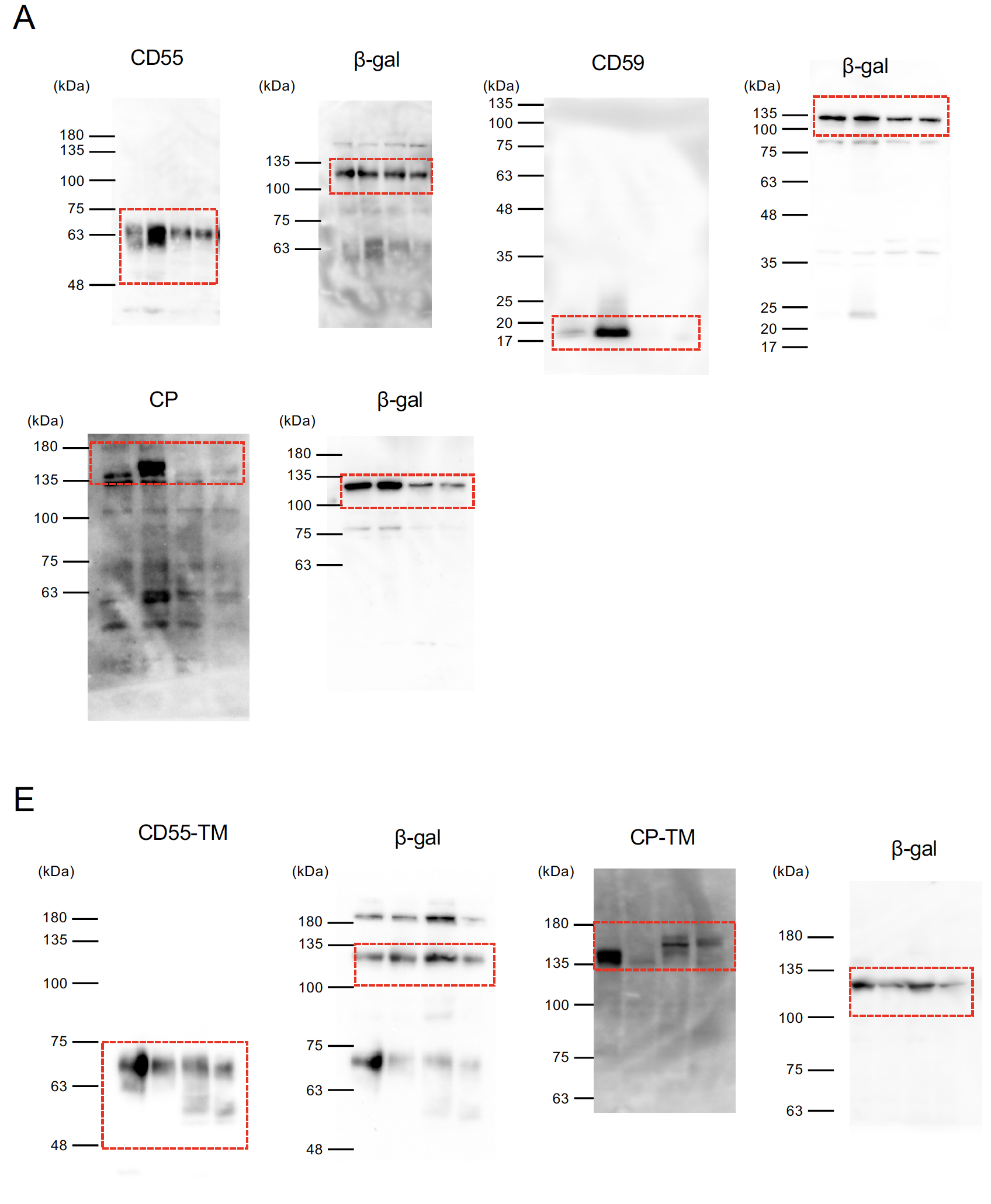
**

**Figure S9. Full-length immunoblot images used in Figure 3.** The panel used is boxed. The same blot was used sequentially (after stripping) for detection in each composite figure. The molecular weights of the marker proteins are indicated on the left of the immunoblot images.

**
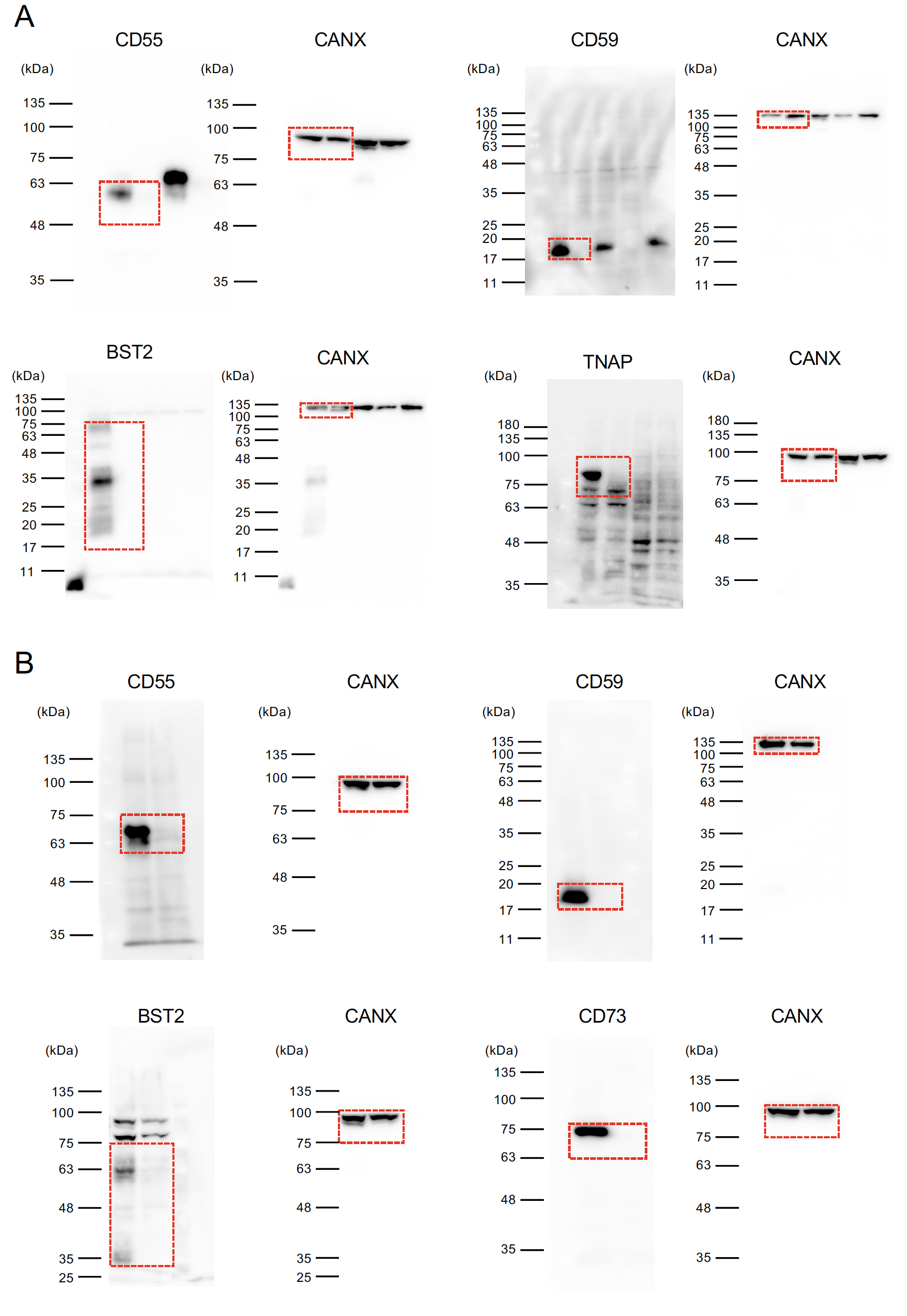
**

**
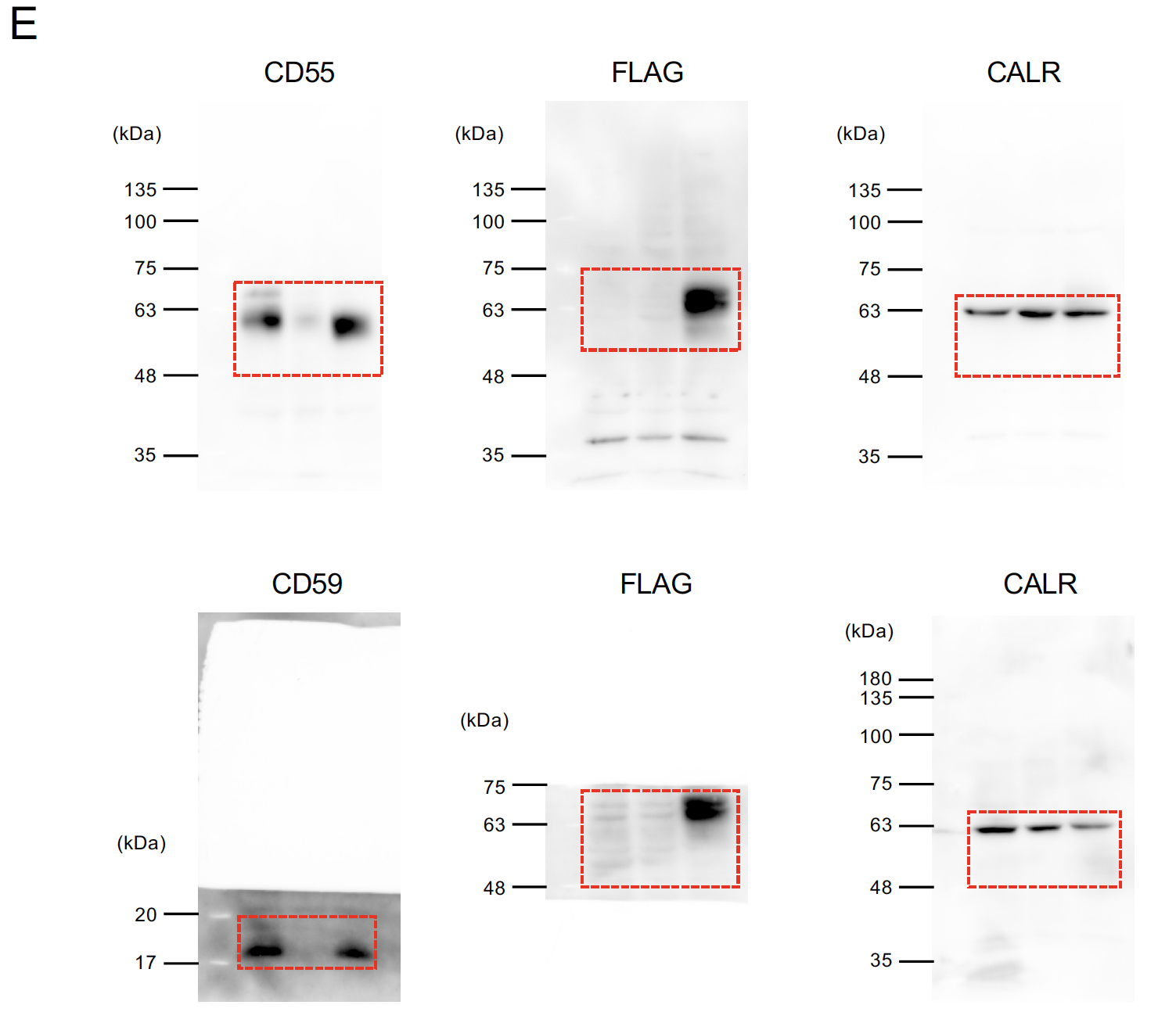
**

**Figure S10. Full-length immunoblot images used in Figure 4.** The panel used is boxed. The same blot was used sequentially (after stripping) for detection in each composite figure. The molecular weights of the marker proteins are indicated on the right of the immunoblot images.

**
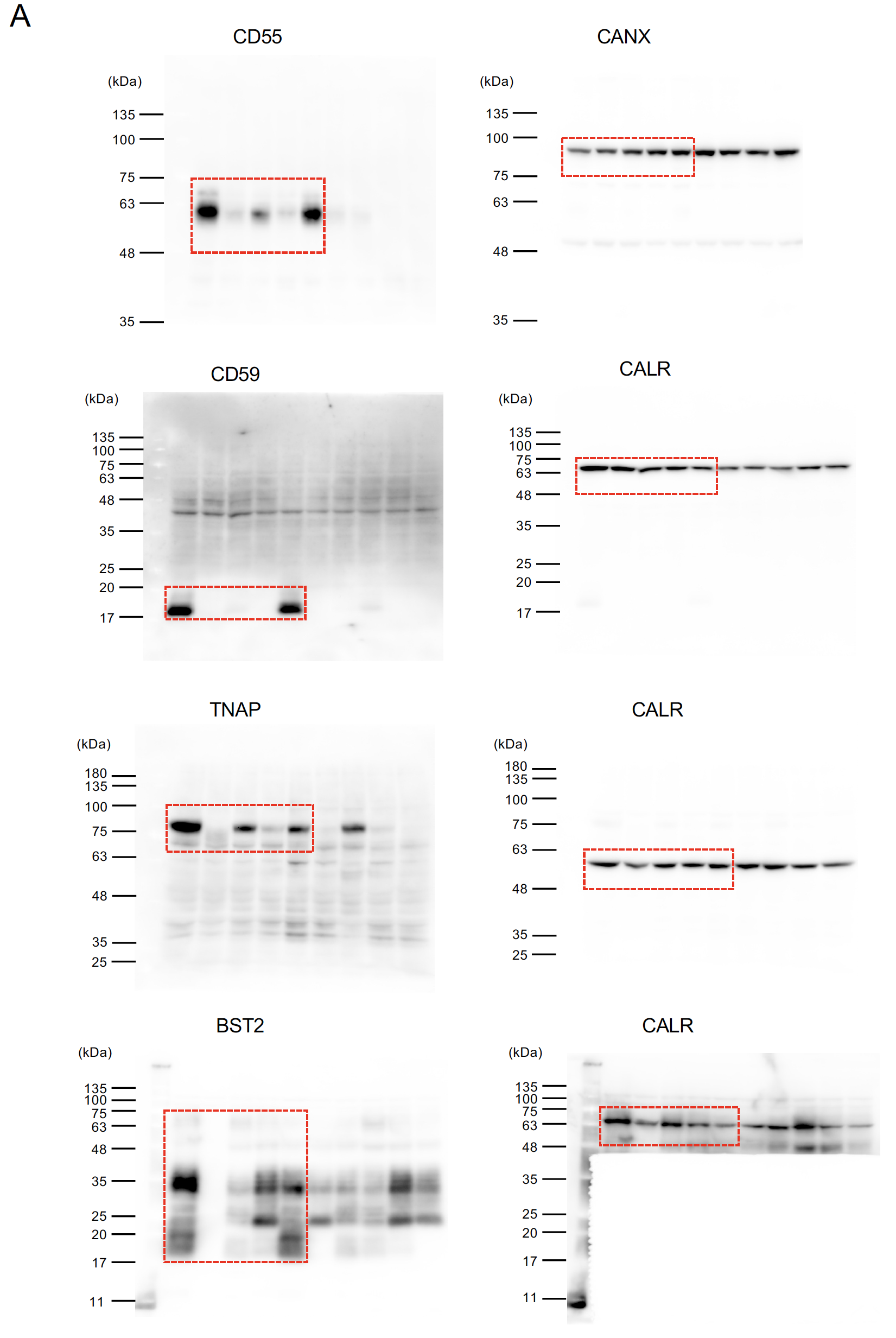
**

**
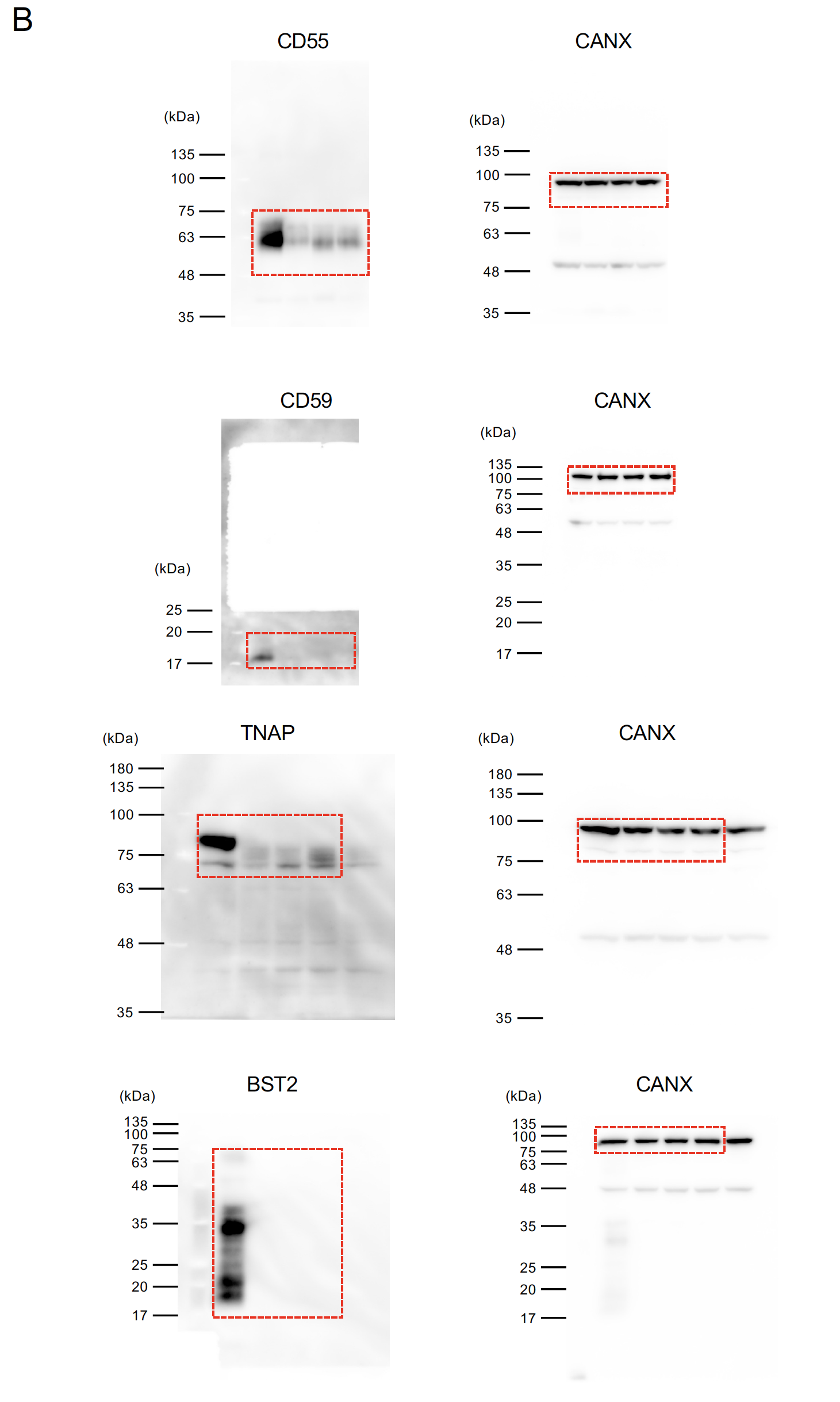
**

**
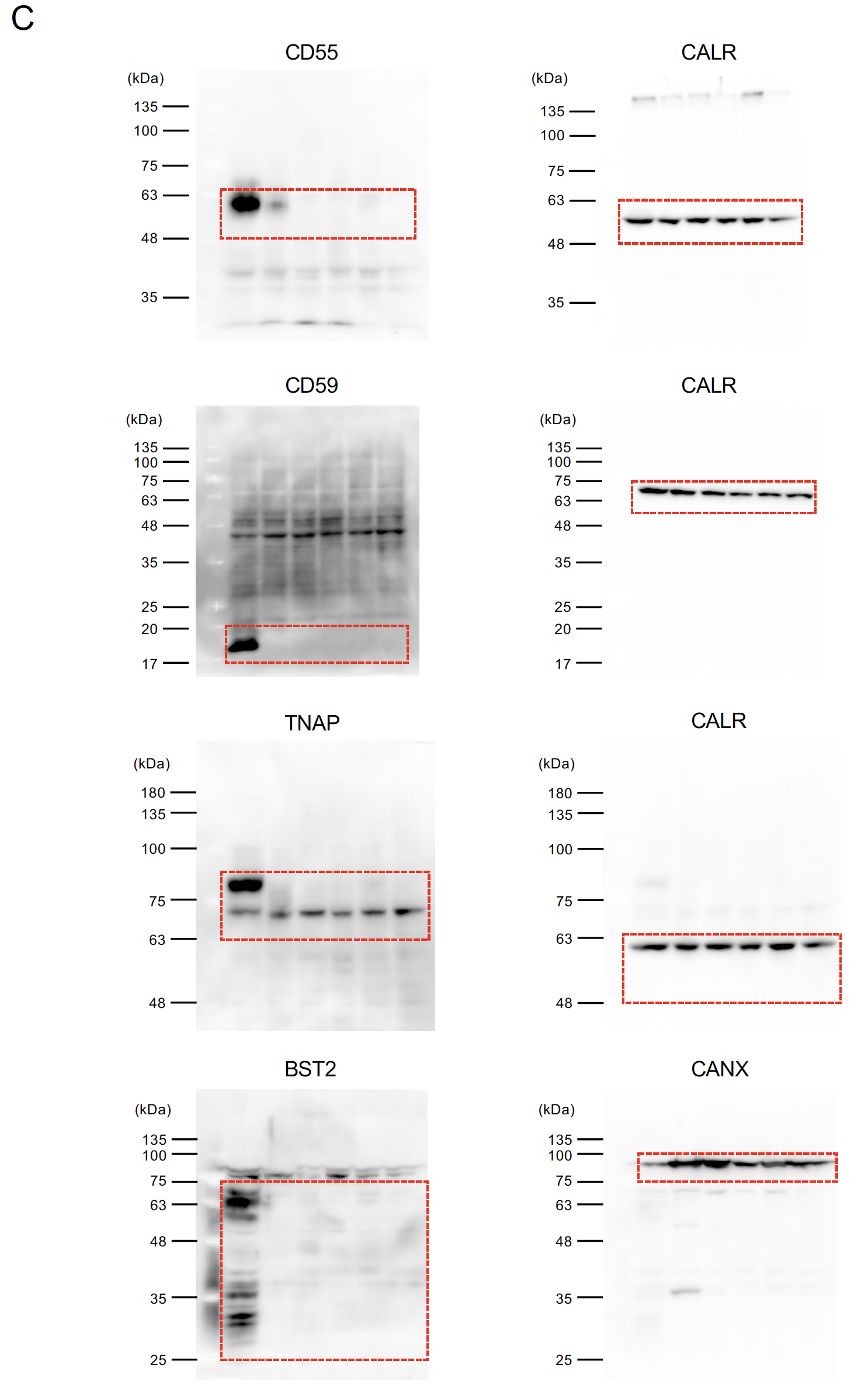
**

**Figure S11. Full-length immunoblot images used in Figure 5.** The panel used is boxed. The same blot was used sequentially (after stripping) for detection in each composite figure. The molecular weights of the marker proteins are indicated on the right of the immunoblot images.

**
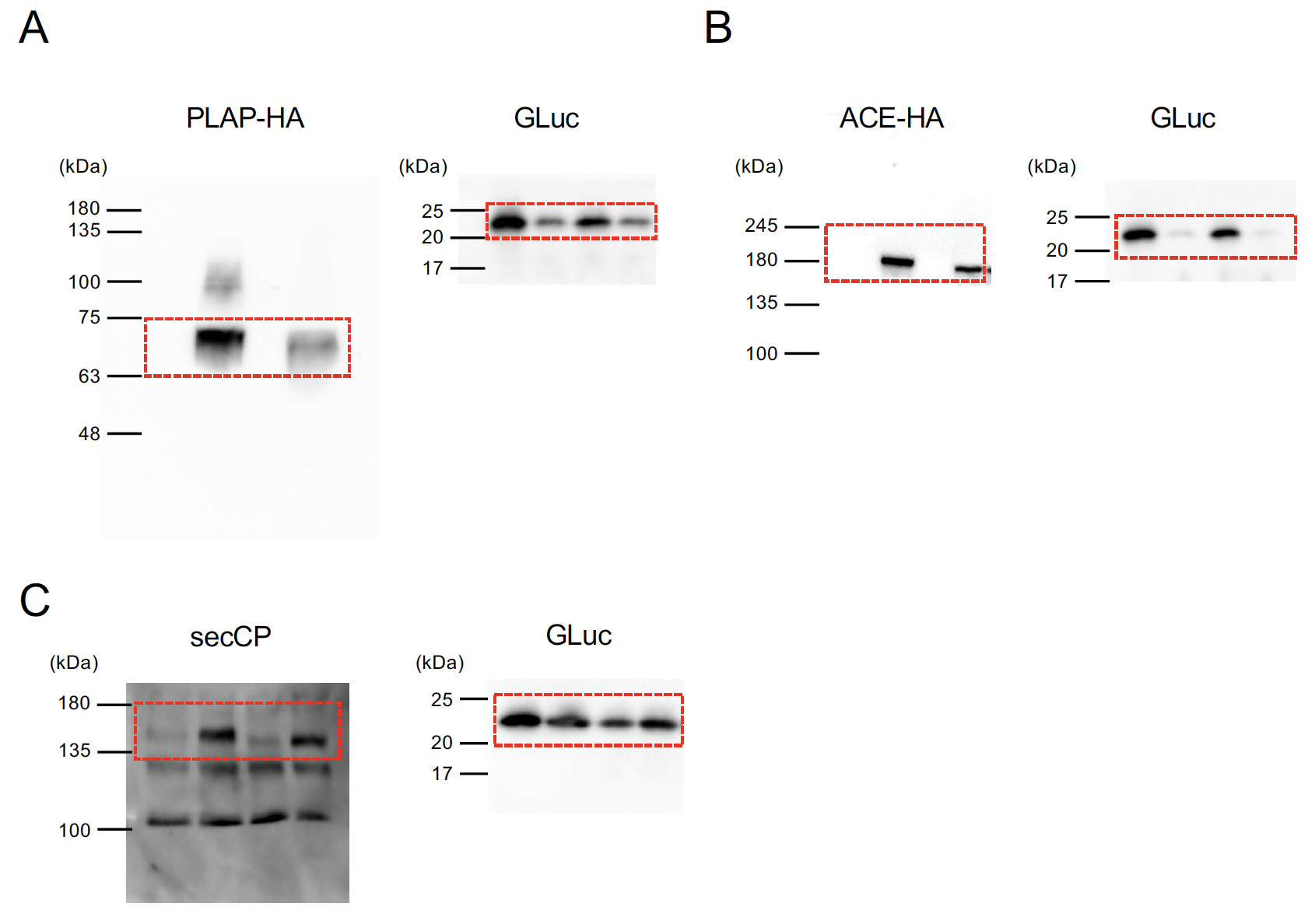
**

**Figure S12. Full-length immunoblot images used in Figure S1.** The panel used is boxed. The same blot was used sequentially (after stripping) or separately for detection in each composite figure. The molecular weights of the marker proteins are indicated on the left of the immunoblot images.


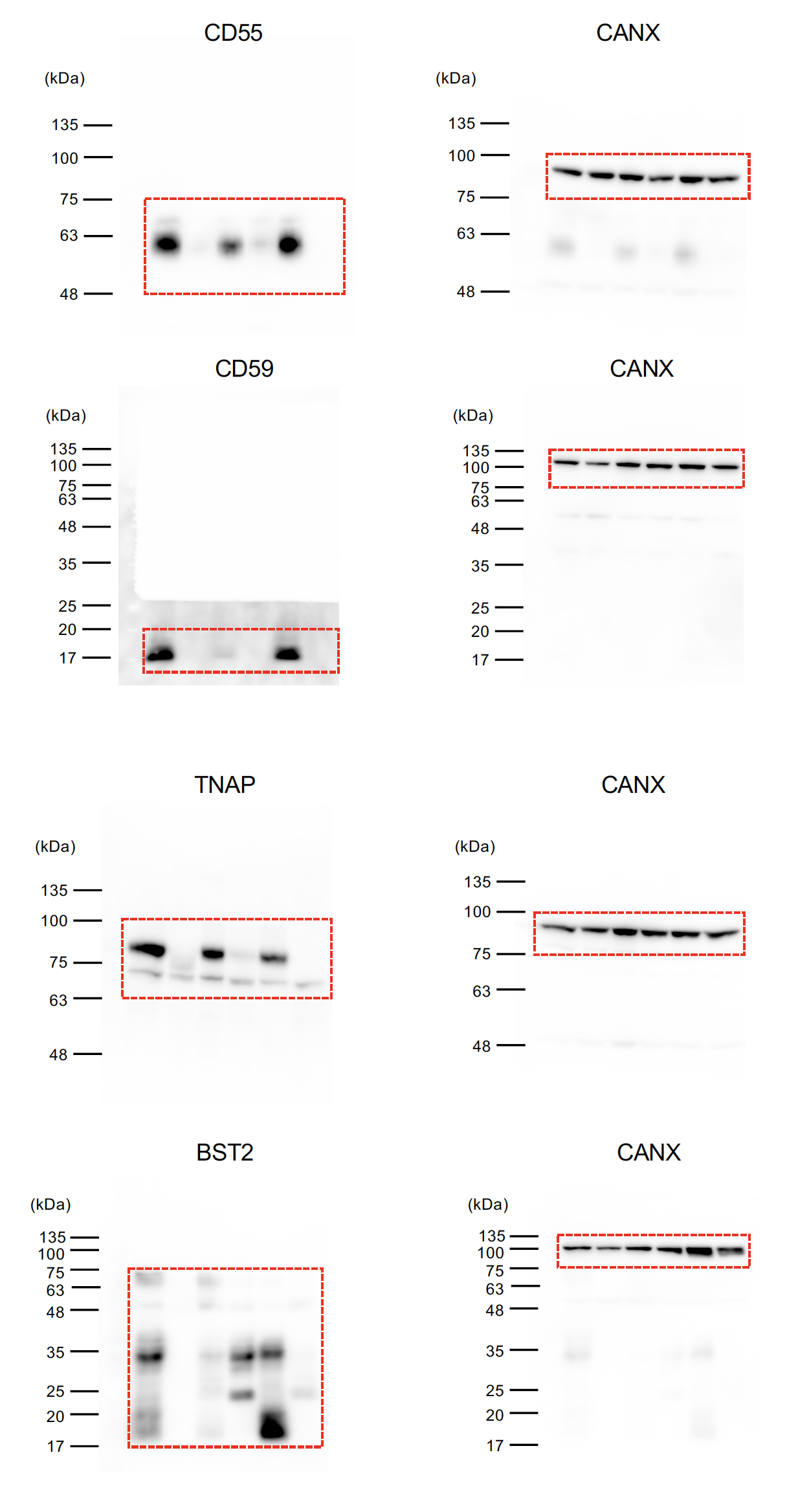


**Figure S13. Full-length immunoblot images used in Figure S3.** The panel used is boxed. The same blot was used sequentially (after stripping) for detection in each composite figure. The molecular weights of the marker proteins are indicated on the right of the immunoblot images.

**Movie S1.** Movement of WT medaka (A), when touched with a fine needle (B).

**Movie S2.** Movement of *Znt*5^+/-^;*Znt7*^+/-^ medaka (A), when touched with a fine needle (B).

**Movie S3.** Movement of *Znt5*^+/-^;*Znt7*^-/-^ medaka (A), when touched with a fine needle (B).

**Movie S4.** Motionless of *Znt5*^-/-^;*Znt7*^+/-^ medaka (A), when touched with a fine needle (B).

**Table S1. Reliable data of the relative protein abundance between HAP-*Z5Z7*-DKO cells and WT HAP1 cells (refer to the Excel file).**

**Table S2. Oligonucleotides used for the generation of sgRNA expression plasmids.**

| **Gene** | **Forward primer (5’ to 3’)** | **Reverse primer (5’ to 3’)** |
| --- | --- | --- |
| *PIGN* | CACCGCTATCAGAGCTACATGACC | AAACGGTCATGTAGCTCTGATAGC |
| *PIGO* | CACCGGTTACTATGCCTCGAATCA | AAACTGATTCGAGGCATAGTAACC |
| *PIGG* | CACCCGTGCTGATTGCTCACTTCC | AAACGGAAGTGAGCAATCAGCACG |
| *PIGA* | CACCGGATATTTCTGACAGAGTTC | AAACGAACTCTGTCAGAAATATCC |
| *PIGT* | CACCGAAGGGTGGCCCCCAGTATC | AAACGATACTGGGGGCCACCCTTC |

Guide RNA sequences are underlined.

**Table S3. Primers used for genomic PCR to confirm gene editing in KO cells.**

| **Gene** | **Forward primer (5’ to 3’)** | **Reverse primer (5’ to 3’)** |
| --- | --- | --- |
| *ZNT5* | TGTGTGAAATAGGGTTTCTAATGTAAGTGG | TAATCTTTGAGCTTAATTCCATTCAGTATT |
| *ZNT7* | GTGAAGAAAGGAGCATGTGAACTG | CAGAGGTAGGTAACAAAGGTAGGAC |
| *PIGT* | CTAATTCCTGGGTAGAGTTCGGGAT | AGATATGTTATGGTTGGCTTTCGTG |
| *PIGO* | GATCACCTGGTCCATCTGGCTAAGA | GGTGGAAAATGTTAGGCCCTGAAGG |
| *PIGN* | CAGTGGGTATTCATGTCAGCAAAGC | GGAATGGGCTCACCTGGAATTTGAA |
| *PIGA* | ATTGACATCTTCTCCCTCAAGACAA | TGTCAAGGTCTACGTCTTCAAGAAT |
| *PIGG* | CACTTAGGAGCCAGTTCTAACTGGA | CATGGGGCACAAATGATGGGTCTCA |

**Table S4. Primary antibodies used in immunoblot (IB) analysis and immunofluorescence (IF) staining.**

| **Antibody** | **IB (dilution)** | **IF (dilution)** | **Catalog No.** | **Source** |
| --- | --- | --- | --- | --- |
| anti-TNAP | 1:3,000 | 1:1,000 | sc-166261 | Santa Cruz Biotechnology, Santa Cruz, CA, USA |
| anti-PLAP | 1:3,000 | 1:1,000 | sc-47691 | Santa Cruz Biotechnology, Santa Cruz, CA, USA |
| anti-CD73 | 1:3,000 | 1:1,000 | 13160 | Cell Signaling Technology, Beverly, MA, USA |
| anti-ACE for sACE | 1:3,000 | 1:1,000 | sc-20791 | Santa Cruz Biotechnology, Santa Cruz, CA, USA |
| anti-ACE for gACE | 1:3,000 | 1:1,000 | BS3485 | Bioworld Technology, Inc, Louis Park, MN, USA |
| anti-ACE2 | 1:3,000 | 1:1,000 | A20069I | BioLegend, San Diego, CA, USA |
| anti-ACE2 | 1:3,000 | - | sc-390851 | Santa Cruz Biotechnology, Santa Cruz, CA, USA |
| anti-CD55 | 1:3,000 | 1:1,000 | sc-51733 | Santa Cruz Biotechnology, Santa Cruz, CA, USA |
| anti-CD59 | 1:3,000 | 1:1,000 | sc-133170 | Santa Cruz Biotechnology, Santa Cruz, CA, USA |
| anti-CP | 1:3,000 | 1:1,000 | MBL137 | MBL Nagoya, Japan |
| anti-BST-2 | 1:3,000 | 1:1,000 | 13560-1-AP | Proteintech Group Inc., Chicago, IL, USA |
| anti-β-galactosidase | 1:3,000 | - | M203-3 | MBL Nagoya, Japan |
| anti-*Gaussia* luciferase | 1:3,000 | - | E8023S | New England Biolabs, Inc., Ipswich, MA, USA |
| anti-HA | 1:3,000 | 1:1,000 | MBL-561 | MBL Nagoya, Japan |
| anti-FLAG M2 | 1:3,000 | - | F3165 | Sigma, St. Louis, MO, USA |
| anti-DYKDDDDK | - | 1:1000 | 20543-1-AP | Proteintech Group Inc., Chicago, IL, USA |

**Table S5. Reliable data of four GPI anchor components (BST2, TNAP, GPC4 and CD55) using more peptides (refer to the Excel file).**

**Table S6. Primers used to generate *Znt5* and *Znt7*-KO medaka.**

| **Amplicon**  **Amplicon** | | **No.** | **Name** | **Sequence** |
| --- | --- | --- | --- | --- |
| *Znt5* | LHA | 1 | Znt5LHA-FW | GAGGctcgagTCAACCCTACTGAGCACGTGCTGTCCGGTG |
|  | LHA | 2 | Znt5LHA-RV | CACTTGATGTAGCTTGGGCCGAAACCGTCAGAGATGAGTC |
|  | RHA | 3 | Znt5RHA-FW | TCAGGGGGAGGTGTGATATCATGCTCCGCTCTGGTCCTGGGC |
|  | RHA | 4 | Znt5RHA-RV | GAGGactagtCCAAACGACTTCAGAGGGGAAGGTTCTTGCACC |
| *Znt7* | LHA | 5 | Znt7LHA-F | GAGGctcgagATAGCTCCAAGATTGCTTGCCA |
|  | LHA | 6 | Znt7LHA-R | CTTGATGTAGCTTGGGCCGAAGGAGTCTGAAATCAGACCT |
|  | RHA | 7 | Znt7RHA-F | TCAGGGGGAGGTGTGATATCACAGGTCTCGCAGCCTCCGTCA |
|  | RHA | 8 | Znt7RHA-R | CGAGGactagtGTATTTTTGAAGACTTTGGTGGCT |
| Cry-mCherry/EGFP-SV40polyAsignal | | 9 | CryPro-FW | GGCCCAAGCTACATCAAGTGACCT |
|  |  | 10 | PolyA-RV | TGATATCACACCTCCCCCTGAACCTG |

Lower letters in sequence indicates the restriction enzyme recognition site.

**Table S7. Sequence information used for editing *Znt5* and *Znt7*-KO medaka.**

| **Gene** | **Name** | **Sequence** |
| --- | --- | --- |
| *Znt5* | Target | AGTCTGGGACTCATCTCTGA**CGG** |
|  | Left homology arm | ACTGAGCACGTGCTGTCCGGTGGCGTGATGGTCAGCGCCGTCTTCTTCCTTATGTGTAAGTGACAGGAAGTGACGCGGCCGTTTCTGCAGCGTGACGACATTCCAACCCAGCTGTGTGACCTTTCCACAGCCTCCAGCATTCTAGCAGCTCCCTCAAAGATGGGGCAAAAAGGCACCTTGGTGGGGTACTCCCCTGAGGGAACGCCCCTGTATAACTTCATGGGGGACGCGCTGCAGCACACGTCCCAGTCGCTGCCGCGCTTCATCAAAGATTCGCTCAAGCAGATCCTGGAGGAGTACGACTCCAGACAGATCTTCTATTTCCTGTGCCTGAATCTGGTACGTGTGTGTTTGTCAGGGGAAATACTTTTACAGCTTCTAGTTCAGGGGACGTGGCAGTTATGGAACCGCATCTGCTTTCAGGCCTTCACCTTCGTGGAGCTGTTTTATGGCGTTTGGACCAACAGTCTGGGACTCATCTCTGACGGTTTC |
|  | Right homology arm | CTGTTCGCTGCCCTCATGACCCGCTGGAAAGCCACCAGGATCTTCTCCTATGGGTGCAGTCTCCTTCACTACGCGGGGCAGCAGCCCGTCAGCTAGAGCTCCCAGACTGACGGTCAAACTGTTTTCCAGGTTTGGTCGAGTGGAAATTCTCTCTGGGTTCATCAACGGCCTGTTCCTGATGGTCATCGCCTTTTTCGTGTTCATGGAGTCCGTCACGCGTTTGTTGGATCCTCCGAACATCAACACAGACATGCTGACGGTCAGTTTCGCCGAGCTCCGTCTGACATCCCAGCAGCTGCAGGAGCAGAGCTGTCACGTCTGTTTATCGGACGTCCGGTTTAAAACAAGAATAAAAGAGTGACGCCGTTCAGCAGCCCTTCACCGATCTACTTCAGGACTTGAACATAGTCAGAGTAGAAGTTTGTCTAACATCATCAGGGGGACTGTTTCAGGTTTTAGGTGCAAGAACCTTCCCCTCTGAAG |
| *Znt7* | Target | GCAGTCAAAGAACATGTGGA**AGG** |
|  | Left homology arm | TTTTTGTTTCACCGCTAATGTGAGGTTGGGGTCGTGAGGGGCTGTAGGCTAGCGGGAGAAAGTGTaAATGGAAATGGGGGCGGGATTCTTCTTCACTAATAGTCCcGCCTACAATTCAGAAGTGAATTTtTAATTTTTCCTGAAACTAATTCTTCCAGAAACTATGTTTAAAAAAACACAGGGTTTTGGGCTAAAAACTCcATAAACATCAATTAAGAAGCACTGGGAaTGCTTTAACAATACATCAAAAGAAGATTGGAGTGGGTCTTTAACCATCTCATCTTCAtCACACTTTTGTGTTTAGTCCTGAAGGATCGTCaGCTGTTTTCCTGTTCATaCTGGTGTTGGACTCTTGCATTAGCAAGTTTTCTTCTCCGTGTTgTCCAtctgtcTCCTTACACGTCTGCTGCTGTGTGTTTGTGTCCAGTTTAGGTCTGATTTCAGACTCCTTC |
|  | Right homology arm | TCTCCAGGTGGAGGTCCAACGACAGCTTCTCCTACGGGTGAGACCTCCTCAACTCTCTGCGCTGTCTCTGGACTCGGTCAAGCCCGGGTCTGATCTTTGCGTGTTCATTGGGTTTCAGGTACGTGAGGGCAGAGGTTCTGGCGGGCTTCGTCAACGGACTCTTCCTCATCTTCACAGCTTTCTTCATCTTCTCTGAAGGAGTCGAGGTGACATTTAGCCTTCACCTgTCTGTATCAGAGGGATCAGACTGATTTTCTGAACATTTCCAAAGACTTTAGTGTAAATATTgAGGAATAACGAAGACAATATTATATTTTTCAAAGCCACCAAAGTCTTCAAAAATAC |

Bold letters in Target sequence indicate PAM sequences.

**Table S8. Primers used for PCR amplification and DNA sequencing to confirm the genotypes of medaka.**

| **Gene** | **Forward primer (5’ to 3’)** | **Reverse primer (5’ to 3’)** |
| --- | --- | --- |
| *Znt5* | CTTCTATTTCCTGTGCCTGAATCTGG | CCATAGGAGAAGATCCTGGTG |
| *Znt7* | GTTCATACTGGTGTTGGACTCTTGCA | CCGTAGGAGAAGCTGTCGTTGGAC |
